# A lasso-based model combining miRNA and clinical variables predicts future risk of breast and ovarian cancer

**DOI:** 10.1038/s41598-026-45020-3

**Published:** 2026-03-24

**Authors:** James W. Webber, Laura Wollborn, Sudhanshu Mishra, Stephanie Alimena, Bryanna Testino, Konrad Stawiski, Wojciech Fendler, Dipanjan Chowdhury, Kevin M. Elias

**Affiliations:** 1 Department of Biomedical Engineering, Lerner Research Institute, Cleveland Clinic Foundation, Cleveland, OH USA; 2 Division of Gynecologic Oncology, Department of Obstetrics and Gynecology and Reproductive Biology, Brigham and Women’s Hospital, Boston, MA USA; 3 Harvard Medical School, Boston, MA USA; 4 Dana-Farber Cancer Institute, Boston, MA USA; 5 Department of Biostatistics and Translational Medicine, Medical University of Łódź, Łódź, Poland; 6 Department of Radiation Oncology, Dana-Farber Cancer Institute, Boston, MA USA; 7 Gynecologic Oncology Section, Obstetrics and Gynecology Institute, Taussig Cancer Institute, Cleveland Clinic Foundation, 9500 Euclid Avenue, ND20, Cleveland, OH 44195 USA

**Keywords:** Ovarian cancer, miRNA, *BRCA1*, *BRCA2*, Risk prediction, Lasso, Gynaecological cancer, Tumour biomarkers

## Abstract

Hereditary breast and ovarian cancer syndrome (HBOC) is principally caused by germline mutations in *BRCA1* and *BRCA2*. However, most women with HBOC are undiagnosed, and some patients meeting clinical criteria for HBOC will have no identifiable mutation after genetic testing. Here, we deploy a lasso-based model to combine serum miRNA profiles with clinical data to identify women at elevated risk for ovarian cancer among a population of 1831 individuals enrolled in an institutional biobank. The miRNA and metadata variables are mapped to two-dimensional space using lasso, after which a linear classification model is trained to estimate “*BRCA*ness” and long-term risk of cancer. After tenfold cross-validation, the method offers a *BRCA* prediction AUC score of 0.98 (95% CI 0.94–1.0) and generalizes across subgroups stratified by age, cancer history, and racial/ethnic group. To demonstrate the clinical relevance of this phenotype, we use the lasso-based model to assess 5-year ovarian cancer risk among an independent cohort of 1044 subjects agnostic to genetic testing results enrolled in a randomized clinical trial. In this unselected population, the output of the lasso-based model strongly correlates to the log 5-year relative risk of ovarian cancer (R = 0.93, 95% CI 0.83–0.97, *p* < 0.0001). When the model was used to predict future onset of ovarian cancer directly, the AUC offered was AUC = 0.75 (95% CI 0.70–0.78). Together, these data suggest the proposed model is a predictor of future ovarian cancer risk.

**Supplementary Information:**

The online version contains supplementary material available at 10.1038/s41598-026-45020-3.

## Introduction

Hereditary breast and ovarian cancer syndrome (HBOC) is characterized by markedly increased lifetime risks of breast and ovarian cancer as well as melanoma, prostate, and pancreatic cancers^1,2^. The most commonly mutated genes in HBOC are the DNA homologous recombination (HR) genes *BRCA1* and *BRCA2*, although mutations in a number of other moderate penetrance genes involved in HR can present a similar phenotype^3^. Moreover, many patients meeting clinical criteria for a diagnosis of HBOC have no identifiable mutation. Currently, the only clinical indications for genetic testing for HBOC are a personal history of a HBOC-related cancer, a strong family history of cancer which might suggest HBOC, or a close family relation with a known genetic mutation linked to HBOC^4^. Routine testing for HBOC is not recommended^5^. As a result, only 10% of the estimated 1 million *BRCA1* or *BRCA2* mutation carriers in the United States are aware of their mutation status and likely even lower percentages among the moderate penetrance genes^6,7^. Compounding this lack of awareness, conventional *BRCA1* and *BRCA2* testing panels have historically had poor representation of genetic variants seen in non-white European populations, leading to higher rates of variants of unknown significance (VUS) among non-white European racial and ethnic groups^8^. Moreover, these testing panels provide uncertain information about cancer risk for individuals with no identifiable mutation.

Recently, we presented data indicating that germline *BRCA1/2* mutation carriers without cancer have distinct circulating microRNA (miRNA) profiles from non-carriers^9^. In that report, a model incorporating 10 miRNAs selected from next generation sequencing (NGS) data achieved 93.9% sensitivity at 80.7% specificity for identifying mutation carriers in an independent validation set. However, NGS remains a resource-constrained technology. Moreover, in clinical practice biomarker profiles are not considered in isolation from patient-specific risk factors. To address these issues, here we combine a targeted miRNA panel with metadata collected from electronic health records to create a new risk-assessment model to predict a “*BRCA*ness” phenotype. We then test the clinical relevance of this high-risk phenotype by using the model to predict the 5-year relative risk of ovarian cancer among presumed average-risk post-menopausal women between the ages of 55 and 75 enrolled in a randomized ovarian cancer screening trial. Our findings suggest that the use of miRNA expression in conjunction with metadata offers significant efficiency and cost benefits as a supplement to conventional genetic testing for *BRCA* mutation using next generation sequencing approaches and may identify additional individuals who should be considered at elevated risk for ovarian cancer.

## Results

### Patient population

Demographic characteristics of the primary study population are shown in Table 1. Study subjects included *n* = 1831 women, the majority of which (95%) lived within a zip code in Massachusetts, receiving routine primary or specialty medical care within a facility affiliated with Mass General Brigham, a large New England healthcare system. Among study subjects, *n* = 100 were known germline *BRCA1/2* mutation carriers and *n* = 1731 were either confirmed non-carriers (*n* = 159) or had not undergone germline testing (*n* = 1572). This population includes 39 subjects with a personal history of an ovarian cancer diagnosis, and 179 with a personal history of a breast cancer diagnosis. The blood samples of the cancer subjects (which were used to measure miRNA expression) were drawn between 1 day and up to 14 years from cancer diagnosis. *BRCA1/2* mutation carriers were more frequently white (96% vs. 79%) and post-menopausal (70% vs. 47%). Not surprisingly, mutation carriers more often had a personal or family history of breast or ovarian cancer. Mutation carriers also had a higher prevalence of benign breast disease. Mutation carriers and non-carriers were otherwise similar in terms of parity, number of abortions, ectopic pregnancies, smoking history, BMI, and gynecologic history. The variables listed in Table 1 were used to train the classification model, other than a personal history of breast or ovarian cancer or a history of oral contraceptive use or hysterectomy. We omitted these latter two variables from the model training due to potential reverse causality. For example, mutation carriers in this study may have undergone hysterectomy as part of risk-reducing surgery to minimize cancer risk or been placed on oral contraceptive pills to reduce ovarian cancer risk.

**Table 1:** Characteristics of the training population.

Variables	Non-carriers	*BRCA* mutation carriers	*p* value
Smoking history (n = 1829)	604 (34.9%)	32 (32%)	0.5493
Obesity (BMI > 30) (n = 1831)	559 (32.3%)	30 (30%)	0.6331
Height (> average) (n = 1829)	705 (40.8%)	38 (38%)	0.5828
Parity (> 0) (n = 1770)	1108 (66.1%)	65 (68.4%)	0.6487
Abortions (> 0) (n = 1426)	309 (23%)	20 (24.7%)	0.7216
Ectopic pregnancies (> 0) (n = 1700)	39 (2.4%)	4 (4.3%)	0.2729
Hormone replacement therapy use (ever) (n = 1690)	219 (13.7%)	14 (15.7%)	0.5848
Oral contraceptive use (ever) (n = 1831)	648 (37.4%)	55 (55%)	0.0004
Tubal ligation (n = 1819)	241 (14%)	10 (10%)	0.2572
Benign gynecologic disease (n = 1831)	347 (20%)	22 (22%)	0.6358
Endometriosis (n = 1802)	196 (11.5%)	15 (15%)	0.2923
Hysterectomy (n = 1826)	254 (14.7%)	36 (36.4%)	< 0.0001
Benign breast disease (n = 1816)	370 (21.5%)	37 (37.4%)	0.0002
Ovarian cancer in family (first degree) (n = 1831)	32 (1.8%)	11 (11%)	< 0.0001
Breast cancer in family (first degree) (n = 1831)	159 (9.2%)	36 (36%)	< 0.0001
Coronary artery disease (n = 1831)	80 (4.6%)	4 (4%)	0.7727
Gallbladder disease (n = 1831)	277 (16%)	16 (16%)	0.9995
Colon polyps (n = 1831)	343 (19.8%)	22 (22%)	0.5949
Hypertension (n = 1831)	576 (33.3%)	29 (29%)	0.3768
Osteoporosis (n = 1831)	193 (11.1%)	19 (19%)	0.0171
Diabetes mellitus (n = 1831)	177 (10.2%)	9 (9%)	0.6933
Personal ovarian cancer history (n = 1829)	25 (1.4%)	14 (15%)	< 0.0001
Personal breast cancer history (n = 1831)	135 (7.8%)	44 (44%)	< 0.0001

The *p* values in the right-hand column indicate if there is a statistically significant difference based on a two-proportion Z-test. In each case, we give the number of subjects belonging to each class and the proportion in parentheses. For some variables (e.g., parity), the data were missing for some subjects. In the first column in parenthesis, we give the number of samples (n) where data were recorded for each variable.

### Classification results

**Fig. 1:**
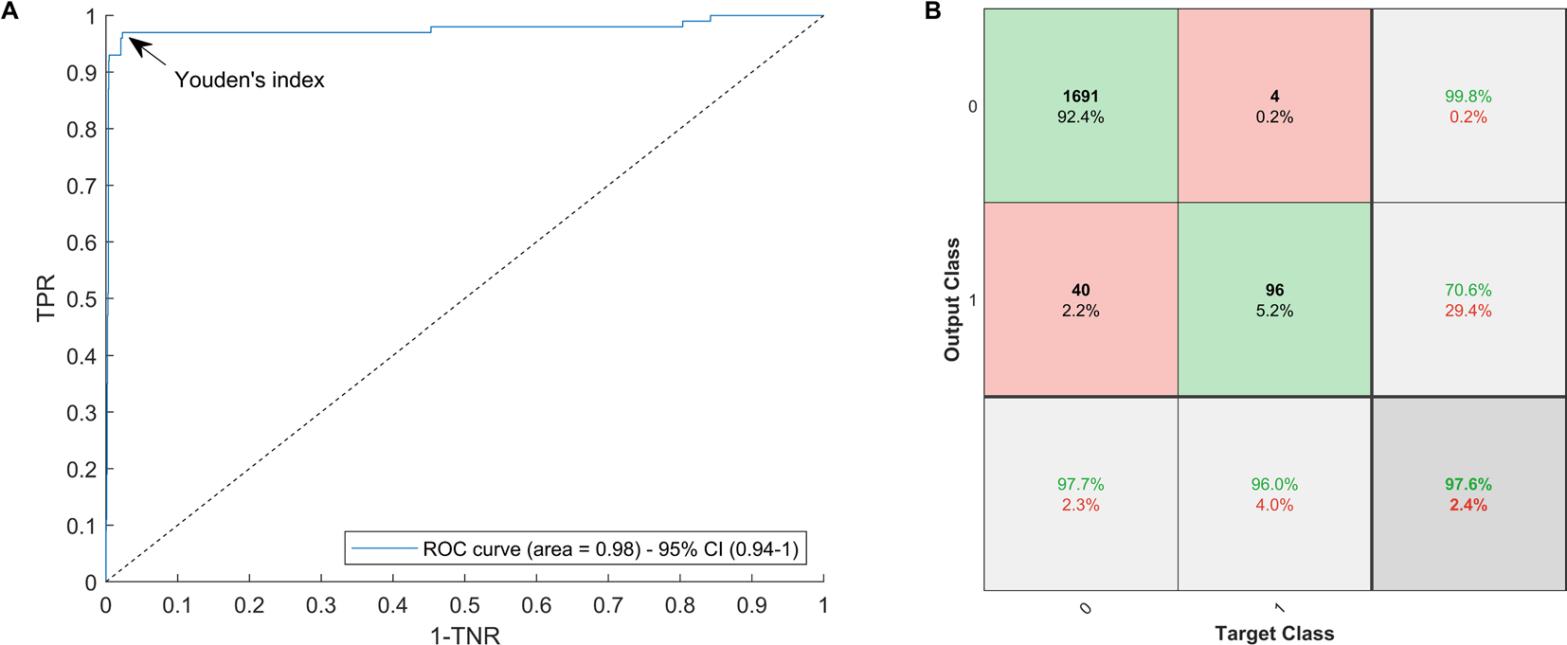
(**A**) ROC curve for *BRCA1/2* classification using the proposed model. (**B**) Confusion matrix using the maximum probability threshold *t* corresponding to the Youden’s index, as highlighted on the ROC curve. In the confusion matrix, 0’s correspond to negatives (i.e., non-*BRCA*), and 1’s correspond to positives (i.e., *BRCA*).

The clinical metadata were combined with miRNA expression data from a focused panel of 179 serum miRNAs using a lasso-based dimension reduction technique to classify study subjects as likely *BRCA1/2* mutation carriers or non-carriers. See Supplementary Table 1 for a full list of all 179 miRNAs considered in this study. After tenfold cross validation (the predictions for all 10 folds were concatenated together and evaluated against the full set of true *BRCA* labels), the area under the curve (AUC) of the receiver operator characteristic (ROC) curve was 0.98 (95% CI 0.94–1.0) (Fig. 1A). The number of non-zero coefficients in the miRNA component of the model was 151 (84% of miRNAs), and the metadata component had 11 non-zero coefficients (58% of metadata variables considered). All miRNA inputted into the model and their lasso weights are listed in Supplementary Table 2. Similarly, the lasso weights of the metadata variables are tabulated in Supplementary Table 3. If the weight is zero, it means the corresponding variable is not used by the model. Conversely, weights with higher absolute value indicate greater relation to BRCAness. For example, the weights corresponding to breast and ovarian cancer family history on the metadata side of the model have larger absolute values relative to the other metadata variables. This makes sense as it is well-known that family history of cancer is related to *BRCA* mutation status.

We classified a subject as a likely *BRCA* mutation carrier if their *BRCA* mutation probability was greater than $t \in \left[ {0,1} \right]$, and conversely as a non-*BRCA* mutation carrier if the probability of *BRCA* mutation was less than or equal to *t*. In Fig. 1B, we present the confusion matrix corresponding to $t = 0.04$, which is the *t* that corresponds to Youden’s index, i.e., the point that maximizes the sum of sensitivity and specificity^10^. The specificity of the model is 98%, the sensitivity is 96%, and the overall classification accuracy is 98%. In this model, 93% of the overall population would be considered low risk of harboring a mutation, while the remaining 7% of subjects, which are more likely *BRCA*1/2 carriers, would be considered at elevated risk. The positive predictive value in this population is 71%, and the negative predictive value is 99.8%.

Using t-SNE plots to visualize the miRNA and metadata components separately, we see the mutation carriers more clearly cluster separately from the presumed non-carriers using miRNAs (Supplementary Fig. S1A,B). However, *BRCA1* and *BRCA2* carriers do not separate from one another using either feature set.

To determine the relative contributions of miRNAs and metadata to the classifier, we compared the lasso-based model (which fuses miRNA and metadata) to the metadata component alone, which would reflect clinical criteria for ordering genetic testing (Supplementary Fig. S2A). After tenfold cross-validation, a metadata only model offers an AUC = 0.68 (95% CI 0.62–0.74), which is markedly lower than the AUC = 0.98 (95% CI 0.94–1) offered by the joint model.

### Effects of varying the number of model input variables

The lasso model retains 151 out of 179 miRNAs when the lasso parameter is selected using cross-validation. Although this is considerably fewer than the number of miRNAs produced by NGS, nonetheless this is still an excessively large number of miRNA features for clinical implementation. We therefore tested whether a smaller set of miRNAs can retain this performance.

**Fig. 2:**
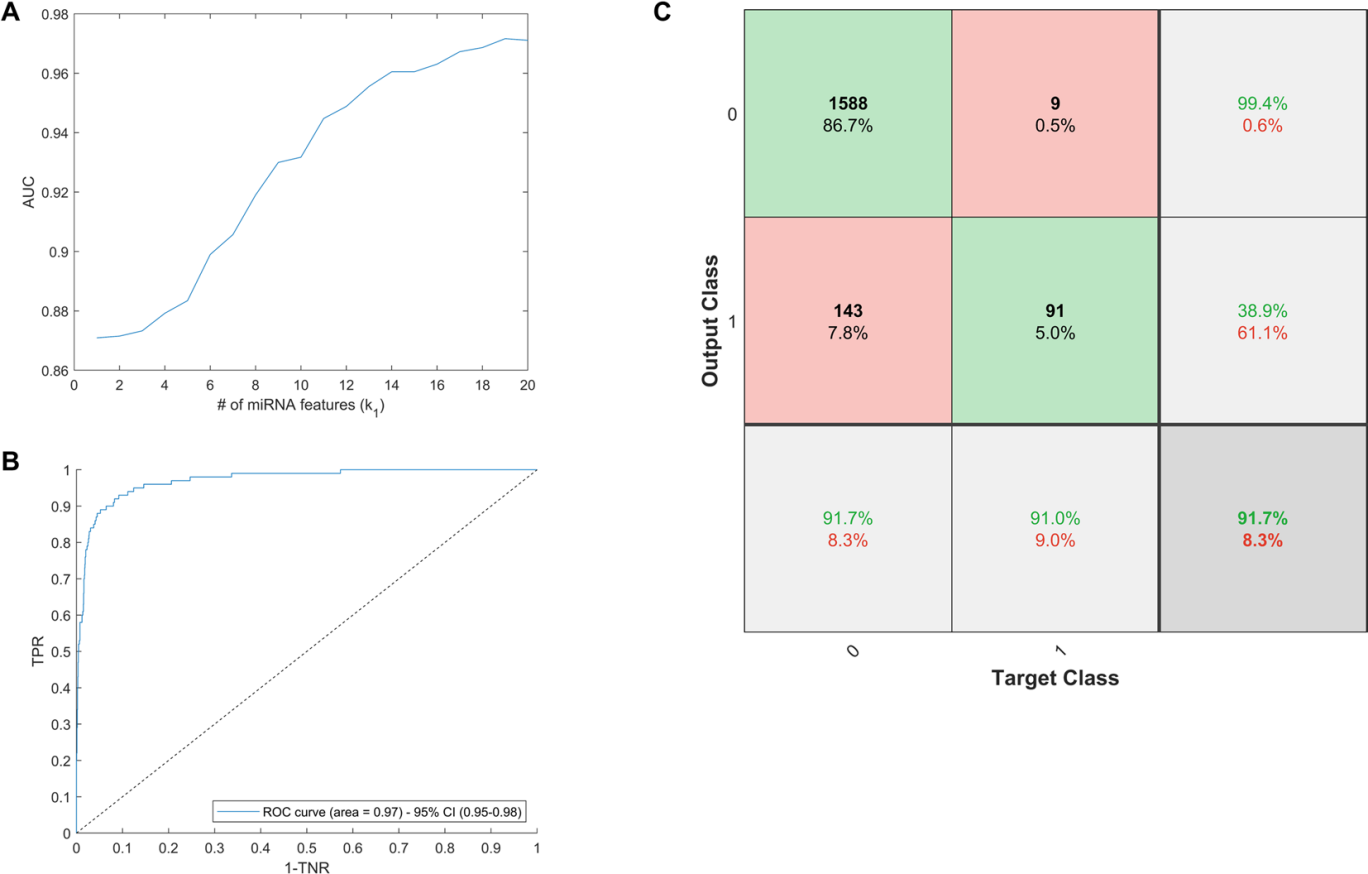
(**A**) Classification AUC with varying numbers of miRNA ($1 \le k_{1} \le 20$), with $k_{2} = 5$. (**B**) ROC curve for *BRCA* prediction results with 20 miRNA and 5 metadata features. (**C**) Confusion matrix for *BRCA* prediction results with 20 miRNA and 5 metadata features. In the confusion matrix, 0’s correspond to negatives (i.e., non-BRCA), and 1’s correspond to positives (i.e., BRCA).

To this end, we examined the effect on model performance if we vary the number of miRNA inputs ($k_{1}$), and metadata features ($k_{2}$). We examined a subset of $k_{1} \le 20$ miRNA and $k_{2} \le 20$ metadata features (selected from Table 1) based on strong linear relation to *BRCA* status. To do this, we restricted the number of non-zero lasso components, i.e., the non-zero entries of **v**_**1**_ and **v**_**2**_ (see the “Methods” section), to a maximum of $k_{1}$ and $k_{2}$, respectively, and we measured the model performance after tenfold cross-validation for $k_{1} \in \left\{ {1, \ldots ,20} \right\}$ and $k_{2} \in \left\{ {1, \ldots ,19} \right\}$ ($k_{2} = 19$ is the upper limit as this is the total number of metadata features used to train the lasso model). In terms of AUC score, we do not see an improvement in performance as $k_{2}$ increased past $k_{2} = 5$, while the AUC increases monotonically with $k_{1}$. In Fig. 2A, we show how the model performance varies in terms of AUC with respect to changes in $k_{1}$, while holding $k_{2} = 5$. The AUC score is maximized at AUC = 0.97 (95% CI 0.95–0.98) when $k_{1} = 20$ and $k_{2} = 5$. The $k_{1} = 20$ miRNA and $k_{2} = 5$ metadata features chosen by the lasso model are listed in Supplementary Tables 4 and 5, respectively. In Fig. 2B, we plot the ROC curve corresponding to the $k_{1} = 20$ miRNA and $k_{2} = 5$ metadata feature model (as above, for comparison Supplementary Fig. S2B compares the joint model to a model using the $k_{2} = 5$ metadata components alone). Figure 2C presents the confusion matrix corresponding to the Youden index in Fig. 2B. Thus, by setting limits of $k_{1} = 20$ and $k_{2} = 5$, we sacrifice little performance in terms of AUC. In this case, the specificity offered by the model is 92%, and the sensitivity is 91%, with overall accuracy of 92%. The positive predictive value of the model is 38.9% with a negative predictive value of 99.4%.

Among the subjects considered in this study, 259 out of 1831 had formal genetic testing for *BRCA* mutations, while the untested subjects were presumed non-*BRCA* mutation carriers when training the lasso model. We considered whether this could create bias or confounding effects in the classification results, especially by misclassifying true positives as presumed negatives. To address this, we considered a subgroup analysis of the performance of the lasso-based model of Fig. 1 as well as just the metadata component if we limited the analysis to mutation carriers and control subjects with known genetic testing results (Supplementary Fig. S2C). The full data model performance is largely retained (AUC = 0.96, 95% CI 0.92–0.98) among patients with documented genetic testing. In comparison, the comparator model (trained using metadata alone) only offers an AUC = 0.57 (95% CI 0.48–0.63) even among formally tested patients. We see a similar effect using the more limited set of model inputs $k_{1} = 20$ miRNA and $k_{2} = 5$ as well (Supplementary Fig. S2D).

### Performance of a BRCA1/2 classifier across race, age, or cancer status subgroups

**Fig. 3:**
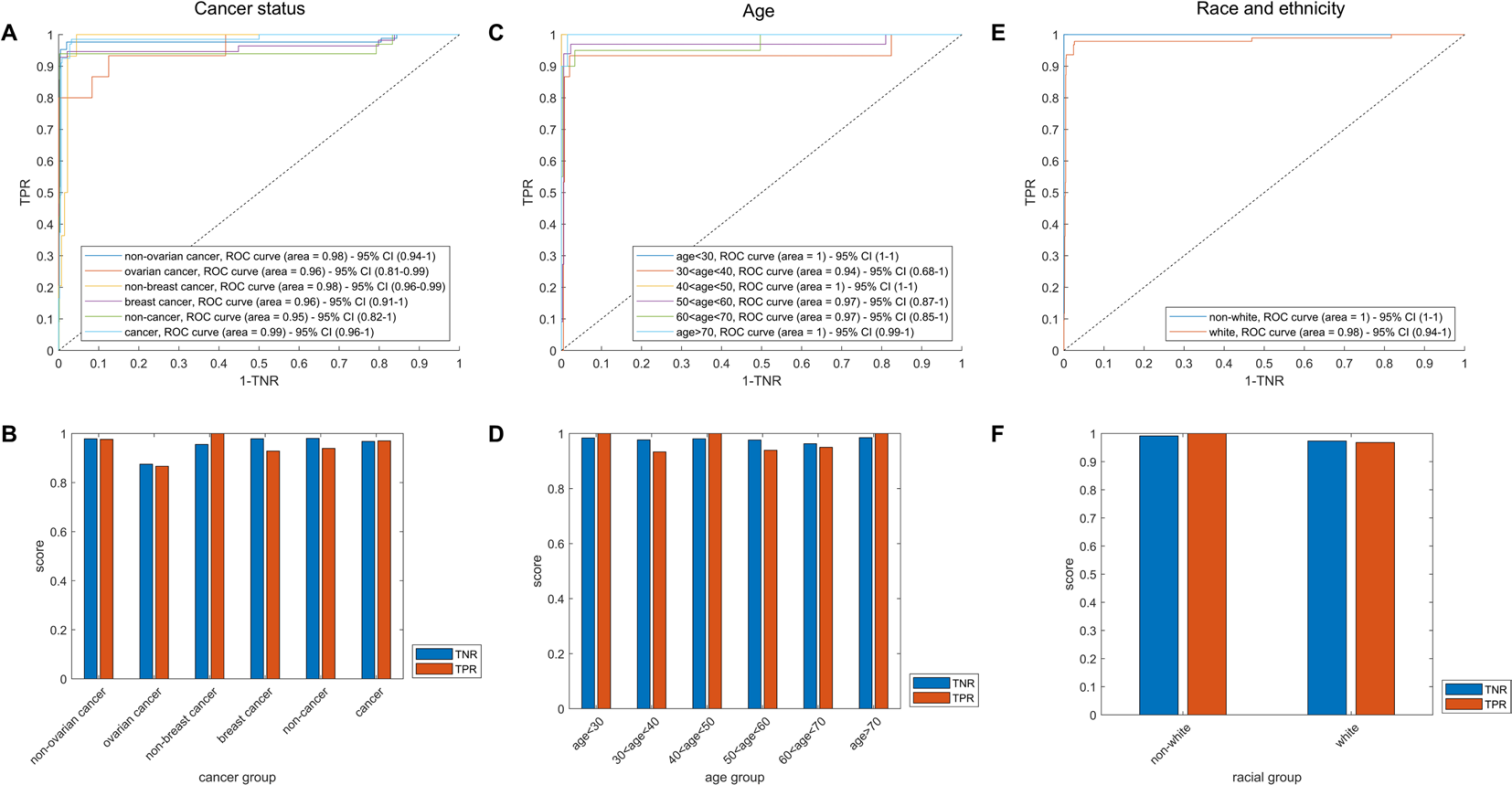
Model performance stratified by (**A**,**B**) cancer history (first column); (**C**,**D**) age (second column); and (**E**,**F**) race/ethnicity (third column) subgroups. Top row: ROC plots. Bottom row: sensitivity (True positive rate—TPR) and specificity (True negative rate—TNR) scores among each subgroup using the *t* = 0.04 *BRCA* probability threshold as in Fig. 1.

To determine the stability of the model across different population subgroups, we performed subset analyses in which study subjects were grouped by race and ethnicity, age, or cancer status. No differences were seen whether the subjects were split by ovarian or breast cancer history, nor if using a collective “cancer” classifier that also includes non-HBOC cancers such as thyroid, cervix, colon, and skin cancers (Fig. 3A,B). Likewise, if we divided the study cohort into 10-year blocks by age, the model performs similarly well among all age groups (Fig. 3C,D). Finally, we examined the impact of race and ethnicity on model performance. Due to small sample sizes for individual minority groups, race was split into “non-Hispanic white” or “all other.” The model performed similarly across racial and ethnic groups (Fig. 3E,F). As with the larger model, classification within these subsets remains consistent even when classified using the more limited set of model inputs $k_{1} = 20$ miRNA and $k_{2} = 5$ described above (Supplementary Fig. S3A–C).

### BRCA1/2 classifier analysis among non-carriers with breast or ovarian cancer

**Fig. 4:**
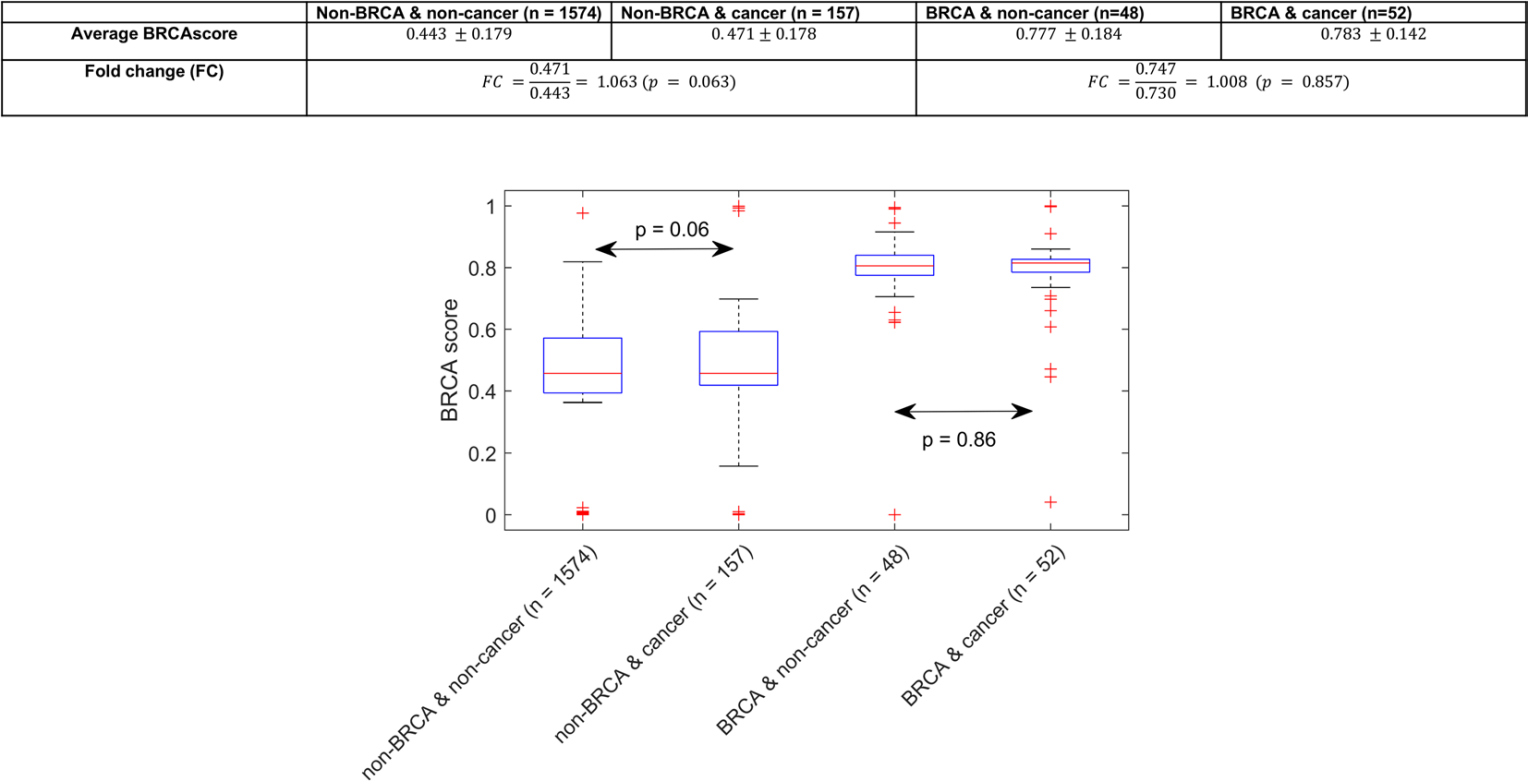
Box plots of mean *BRCA* scores stratified by germline status and history of breast or ovarian cancer. In the box plot, the *p* values indicate whether there is a statistically significant difference in mean *BRCA* score. The table above tabulates the mean and standard deviation values in the box plot and gives the corresponding Fold Change (FC) and *p* values for each comparison. Here, “cancer” means the subject has on record a previous diagnosis of ovarian or breast cancer.

While germline *BRCA1/2* mutations are identified in 13–15% of ovarian cancers and 3% of unselected breast cancers, an additional 5–7% of ovarian cancers and 3% of breast cancers harbor somatic *BRCA1/2* mutations and up to half of ovarian cancers may have some defect in homologous recombination^11–13^. We therefore questioned whether the miRNA profiles of non-mutation carriers with breast or ovarian cancer might more closely resemble mutation carriers with or without cancer. While among all study subjects, non-cancer subjects have a lower mean “BRCAness” score of 0.45 compared to cancer subjects (0.55, *p* < 0.0001), these differences are not significant when stratified by mutation status (Fig. 4). Thus, while this model indicates an increased propensity to develop ovarian cancer, this particular model is not diagnostic for ovarian cancer. Alternately, the model reflects cancer risk but is not inherently biased by incident or prior cancers.

### Biologic implications for the “*BRCAness*” miRNA profile

**Fig. 5:**
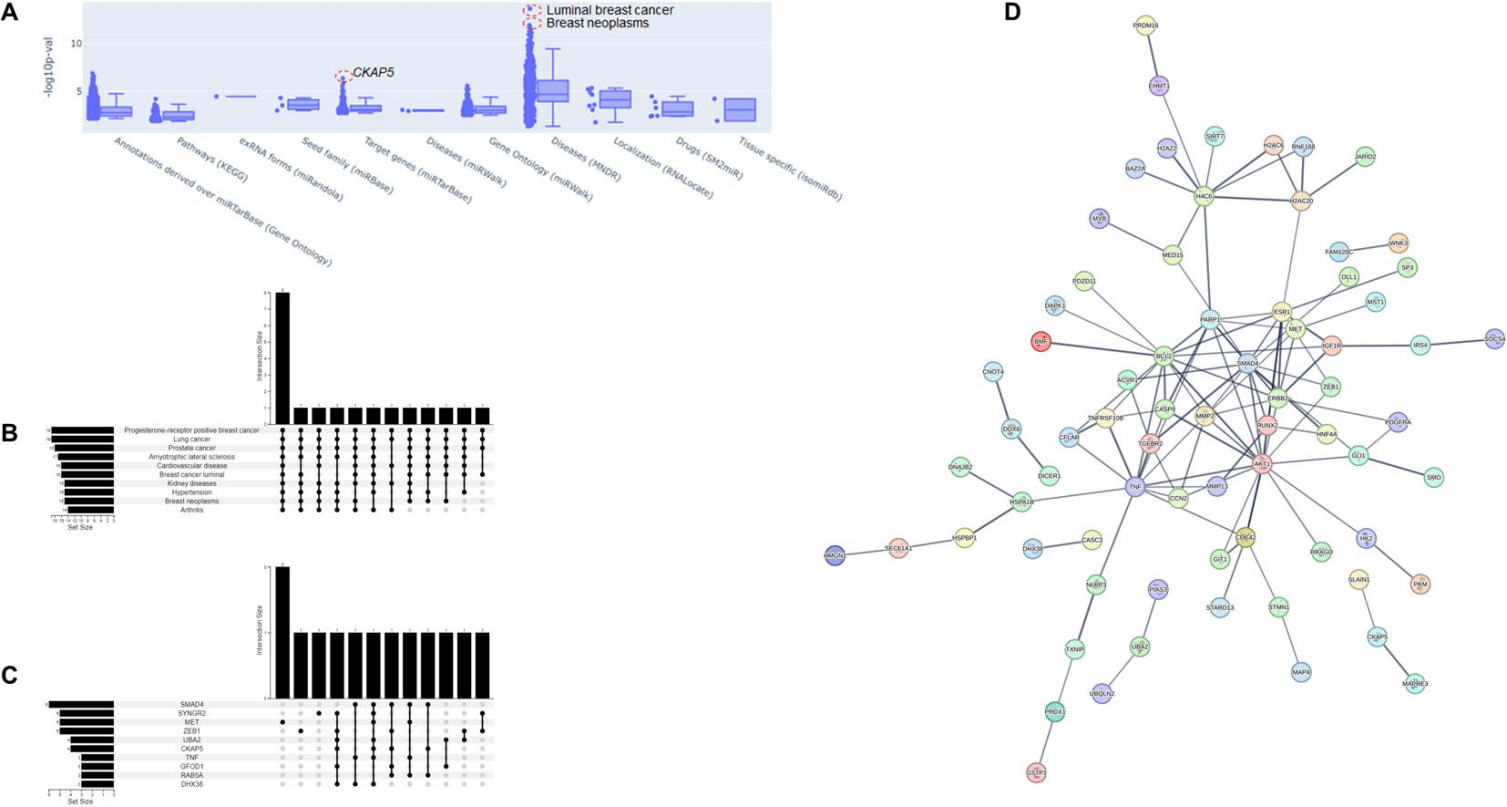
Over-representation analysis with Benjamini–Hochberg adjustment. (**A**) Summary of all categories. (**B**) Upset plot of most significant enriched categories by mammalian ncRNA-disease repository category. (**C**) Upset plot of most significant enriched categories by target genes (miRTarBase). (**D**) Interactome of significant target genes (only linked nodes shown) using STRING v.11.0.

miRNAs are biologically active molecules, and their functions can be predicted based on sequence homology to target mRNAs. Moreover, several databases have emerged which allow categorization of miRNA functions around disease associations and cell signaling pathways. We performed an over-representation analysis to identify possible known correlations with the 20 miRNAs from the limited feature set. The most significant association was with luminal breast cancer and breast neoplasms (Fig. 5A), which is concordant with a miRNA set which correlates with *BRCA1/2* mutations. Looking more closely at disease-related categories, we also noted a strong association with prostate cancer, another *BRCA*-related malignancy (Fig. 5B). Among the predicted target genes of this miRNA set, we identified *SMAD4*, a critical transcription factor for pancreatic cancer pathogenesis, and *ZEB1*, a key regulator of epithelial to mesenchymal transition (Fig. 5C). We also entered the predicted gene targets into a pathway modeling tool, which suggests shared signaling centered around estrogen signaling (*ESR1)*, HER2 (*ERBB2)*, and *PARP1*, all of which are highly relevant for ovarian and breast cancer pathogenesis (Fig. 5D).

### Clinical application: relation between model predictions and ovarian cancer risk

While a blood test which can help identify *BRCA* mutation carriers may be useful to prompt more women to get genetic testing, the main clinical relevance of a *BRCA*ness score is whether this model actually anticipates an increased cancer risk among otherwise presumably healthy individuals. Since *BRCA* mutation carriers are at increased relative risk for certain types of cancer (e.g., breast and ovarian), we hypothesized that the *BRCA*ness score would correlate to relative cancer risk.

When the lasso-based model was used to predict ovarian or breast cancer directly, the model offered 87% classification accuracy with 95% specificity and 28% sensitivity (Supplementary Fig. S4A). The corresponding AUC score was AUC = 0.63 (95% CI 0.59–0.68). The specificity and sensitivity scores, and AUC, are notably lower than in Fig. 1, as we are using a model trained on *BRCA* mutation to predict likelihood of cancer directly. However, this model is not intended as a diagnostic test. When considered as a prognostic risk factor, the *BRCA* score outputted by the model was highly correlated to the log relative risk of breast/ovarian cancer (R = 0.90, 95% CI 0.76–0.96, *p* < 0.0001), indicating the model’s ability to assess long-term cancer risk (Supplementary Fig. S4B). The limited data model performed similarly well (Supplementary Fig. S4C,D). Note, there is some noise in the relative risk curves, and they are not perfectly monotone due to small numbers of cases in some *BRCA* score bins.

**Fig. 6:**
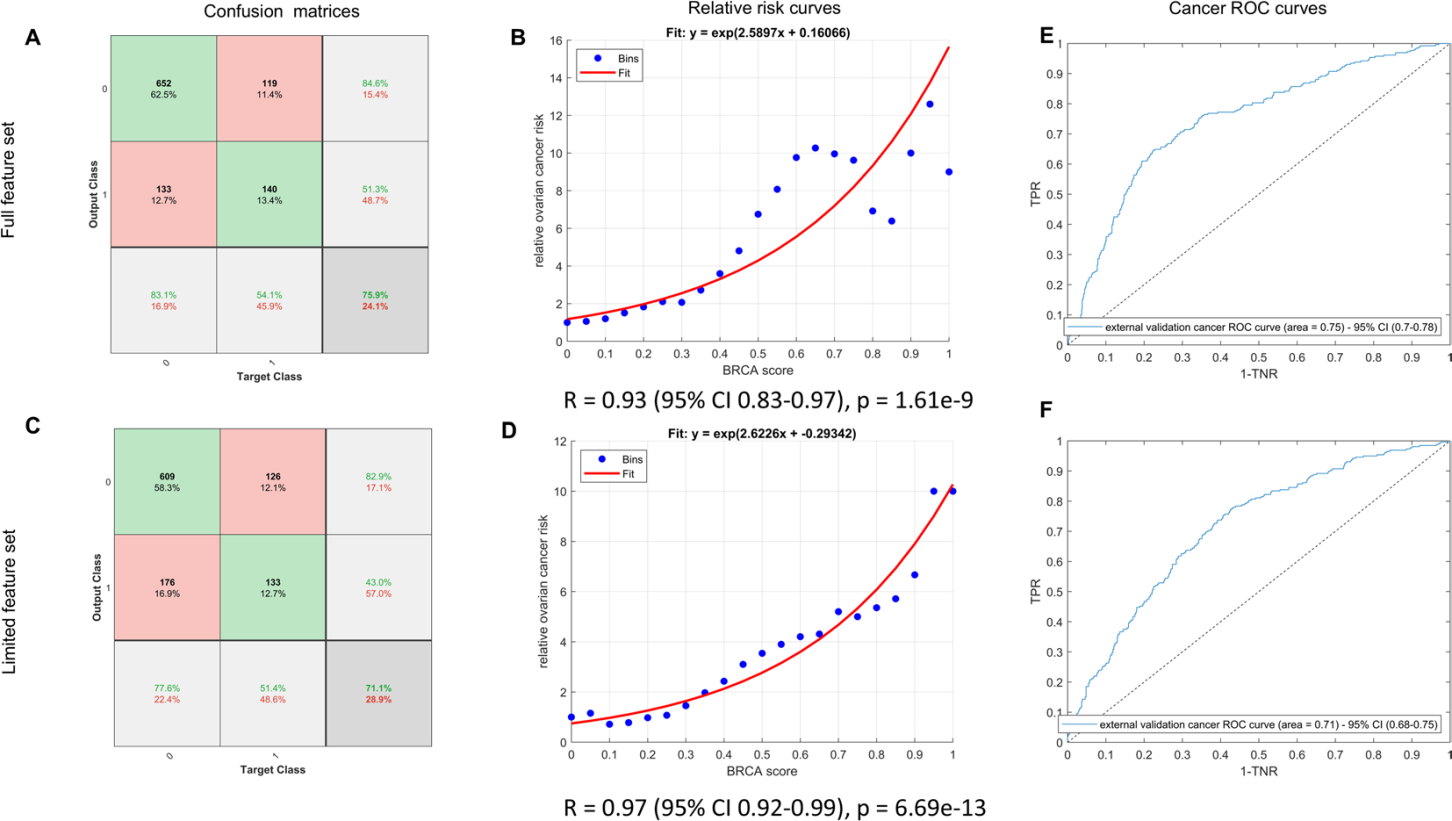
External validation results on PLCO data. Top row: full data results. Bottom row: limited feature model results. The confusion matrices corresponding to the Youden indices on the training ROC curves are given in (**A**) and (**C**). We report the external ROC curves and AUC scores in the right-hand column. The upward trend in log relative cancer risk with *BRCA* score observed in (**B**) has R = 0.93 (95% CI 0.83–0.97, *p* < 0.0001). In (**D**), R = 0.97 (95% CI 0.92–0.99, *p* < 0.0001). The binned relative risk scores are the blue dots and the goodness of fit is shown in red. Note, the relative risk curves become noisier when *BRCA* score > 0.7 due to small sample numbers (i.e., most PLCO subjects had *BRCA* score < 0.7).

To further validate the clinical relevance of the model, and to prevent overfitting, we evaluated how the model would perform in an independent population. To do this, we investigated an external cohort comprised of $n = 1044$ samples collected as part of the PLCO cancer screening trial. Clinical characteristics of the PLCO population appear in Table 2. This population consists of 259 subjects later diagnosed with ovarian cancer and 785 matched controls. The cancer cases had their blood drawn at a range of times between 1 and 1814 days (up to 5 years) before cancer diagnosis. We used the *BRCA*ness score to calculate the cancer prediction accuracy and relative ovarian cancer risk using the thresholds calculated on the training data, and we evaluated both the full and limited data model performance (Fig. 6A–D). The full data model offers 76% long-term cancer prediction accuracy, with 83% specificity and 54% sensitivity, and the limited data model performs similarly (see Fig. 6A,C). The external AUC when predicting ovarian cancer directly was AUC = 0.75 (95% CI 0.70–0.78). There is a strong positive correlation between *BRCA*ness score and the log of the 5-year relative ovarian cancer risk using the full and limited data models (R = 0.93, 95% CI 0.83–0.97, *p* < 0.0001, and R = 0.97, 95% CI 0.92–0.99, *p* < 0.0001, respectively, Fig. 6B,D). This suggests the relative risk of ovarian cancer increases exponentially with the *BRCA*ness score. For example, if a subject presented with a *BRCA*ness score of 0.6, using the limited data model and the fitted curve in Fig. 6D, their 5-year relative risk of ovarian cancer is over threefold, whereas a *BRCA*ness score of 0.9 would indicate a 5-year relative risk of approximately eightfold.

**Table 2:** Characteristics of the PLCO population.

Variables	Non-ovarian cancers (controls)	Ovarian cancer (cases)	*p* value
Smoking history (n = 1044)	346 (44.1%)	112 (43.2%)	0.8147
Obesity (BMI > 30) (n = 1036)	169 (21.7%)	61 (23.6%)	0.5200
Height (> average) (n = 1043)	370 (41.7%)	127 (49.2%)	0.5596
Parity (> 0) (n = 1040)	710 (90.7%)	234 (91.1%)	0.8575
Abortions (> 0) (n = 1040)	250 (31.9%)	67 (26.1%)	0.0767
Ectopic pregnancies (> 0) (n = 1035)	12 (1.5%)	0 (0%)	0.0481
Hormone replacement therapy use (ever) (n = 1043)	517 (65.9%)	188 (72.6%)	0.0477
Tubal ligation (n = 1044)	147 (18.7%)	43 (16.6%)	0.4424
Benign gynecologic disease (n = 1024)	646 (84.1%)	241 (94.1%)	< 0.0001
Endometriosis (n = 1017)	84 (11.0%)	23 (9.0%)	0.3542
Benign breast disease (n = 1026)	209 (27.2%)	73 (28.3%)	0.7365
Ovarian cancer in family (first degree) (n = 1035)	33 (4.2%)	10 (3.9%)	0.8414
Breast cancer in family (first degree) (n = 1035)	146 (18.7%)	42 (16.5%)	0.4383
Coronary artery disease (n = 1044)	34 (4.3%)	14 (5.4%)	0.4741
Gallbladder disease (n = 1043)	130 (16.6%)	36 (13.9%)	0.3063
Colon polyps (n = 1042)	42 (5.4%)	18 (6.9%)	0.3423
Hypertension (n = 1044)	250 (31.8%)	88 (34.0%)	0.5253
Osteoporosis (n = 1041)	85 (10.9%)	11 (4.2%)	0.0014
Diabetes mellitus (n = 1044)	48 (6.1%)	9 (3.5%)	0.1049

The *p* values in the right-hand column indicate if there is a statistically significant difference based on a two-proportion Z-test. In each case, we give the number of subjects belonging to each class and the proportion in parentheses. For some variables (e.g., parity), the data were missing for some subjects. In the first column in parenthesis, we give the number of samples (n) where data were recorded for each variable. We include the variables that were used to train the classification model.

## Discussion

In this work we present a novel tool for identifying a predisposition to breast and ovarian cancer that combines a serum analyte (miRNA expression) with clinical metadata (personal and family history). We used a lasso-based model to reduce the dimensionality of the miRNA and metadata inputs to two dimensions. Following this, a linear classifier was trained on the reduced dimension data to determine “*BRCAness*” based on the similarity of the profile to those of known *BRCA1/2* pathogenic mutation carriers.

This adds to our prior report on unique miRNA signatures for *BRCA* mutation carriers^9^. Whereas in our previous study, we did not investigate how *BRCAness* related to long-term cancer risk, here we specifically tested the ability of the proposed model to assess long-term (5-year) cancer risk on an external population collected as part of the PLCO cancer screening trial. Our primary finding is that the model output has a strong positive correlation with the log relative cancer risk (R = 0.93, 95% CI 0.83-0.97, *p* < 0.0001). The 5-year cancer prediction accuracy offered by the model was 76%, with 83% specificity and 54% sensitivity, which is notable as the majority (92%) of the PLCO subjects considered here had their blood drawn more than one year before cancer diagnosis, and thus likely did not have active cancer at the time of blood draw. We saw similar performance when the model was evaluated internally on the biobank data set, after tenfold cross-validation. This implies the ability of the proposed model to predict future cancer risk.

In terms of *BRCA* prediction, the proposed model offered an AUC score of 0.98 after tenfold cross validation, which shows improvement over the AUC of 0.89 we previously reported using next generation sequencing and may help to improve efficiency of genetic testing for *BRCA* mutations^9^. We also evaluated the model performance across different racial and ethnic, age, and cancer status groups and have shown the model largely retains its predictive capacity, which is important in light of our recent works suggesting these variables can significantly impact serum miRNA expression^14,15^. We also tested the model performance with more limited sets of miRNA and metadata and found that a smaller set of inputs nearly replicated the full data model.

Our approach expands on models based on clinical data which can be used for predicting *BRCA* mutation status^16–18^. One of the goals of the present work is to assess how such clinical factors can be combined with miRNA to improve performance. Models based purely on clinical data offer AUC scores up to AUC = 0.77, which is lower than the proposed model (AUC = 0.98) which combines both data types^19^, although we note that our AUC is the result of cross-validation, whereas the studies in the literature typically use external validation sets. In order to maintain optimal accuracy, some of the models previously reported use very detailed clinical and family history information (e.g., Ashkenazi Jewish ancestry), and personal history of cancer (not just family history), which our model does not rely on^16,20^, although it is noted that the models discussed in the literature do not strictly require such information. The external AUC performance of our model was AUC = 0.75, when used to predict ovarian cancer directly on the PLCO cohort. This is an improvement over models proposed in the literature for ovarian cancer prediction which use only clinical risk factors (similar to, e.g., BRCApro), where the AUC scores typically range from 0.59 to 0.64^21^. While a direct comparison with the models from the literature would be difficult as, e.g., the cohort populations adopt different characteristics, we provide a comparison to a baseline model which uses only the clinical information available as part of our chart reviews. This includes some of the variables used to train the models in the literature, such as family history of cancer, and we find the performance is improved when combining clinical information with miRNA. Of course, we are not suggesting that our model should necessarily replace anything proposed in the literature, but the BRCAness score we present shows merit in assessing long-term ovarian cancer risk based on the results provided. It would be interesting to investigate in further work if the performance could be improved further if more specific clinical factors (e.g., ancestral history) were available in conjunction with miRNA.

The current work is distinct from prior case–control studies, including our own, that use miRNA expression to distinguish healthy subjects, or subjects with benign tumors, from those with cancer^10,22–24^. Such studies do not identify early indicators of cancer risk, as the models are trained on subjects who actively have cancer, most of which are late-stage cancers. As the outputs from the models are binary (i.e., cancer vs. control), they cannot account for competing cancer risks with shared risk factors, such as breast, ovarian, and uterine cancer. In contrast, *BRCA* mutation is a binary output, and thus we do not encounter the same competing risk issue. Identifying these individuals at high risk for cancer who could benefit most from risk reduction surgeries or intensive surveillance strategies thereby provides a different approach to preventing cancer deaths^25^.

Our findings add to the literature demonstrating that *BRCA* mutations are associated with changes in miRNA expression. Tumor profiling has shown that both breast and ovarian cancer tissues from women with germline mutations in *BRCA1/2* are distinct from sporadic tumors^26,27^. In the current work and in our prior report, we show that this relationship extends to circulating miRNA profiles in both healthy women and women with cancer^9^. This suggests a key role for miRNAs in the phenotype of HBOC, which is supported by pathway modeling using the predicted targets of these miRNAs, which cluster around well-defined ovarian and breast cancer signaling pathways. Determining whether the miRNA changes associated with *BRCA* mutations are protective against genotoxic stress or contribute to oncogenesis will be an interesting area for future work.

The current study has several strengths. First, we present a large clinical dataset reflecting the clinical and demographic diversity of women seen in actual clinical practice, as well as data for a focused subgroup of women participating in a cancer prevention trial. This stands in contrast to other miRNA based models from the literature focused on smaller sample sets and trained on miRNA expression from a single cancer type^26^. Second, we describe a *BRCA* mutation prediction model which works equally well among women with and without HBOC-associated cancers. Our data include healthy subjects, and subjects with a variety of cancers, such as breast, ovarian, skin, and cervical cancer. Finally, the use of miRNA expression technology and metadata to predict *BRCA* mutation offers significant efficiency and cost benefits when compared to conventional genetic testing for *BRCA* mutations using next generation sequencing^28^. A first-pass screening based on miRNA and metadata has very high sensitivity, which can narrow down the wider population to a high-risk subgroup which can then be moved on to further screening (e.g., conventional genetic testing).

We also acknowledge some of the limitations of our study. This work focuses on *BRCA1/2* mutations and predicting long-term cancer risk. Insufficient data are available to understand how this model would classify moderate penetrance HBOC genes such as *PALB2* or *BRIP1* or other types of DNA repair defects, such as Li-Fraumeni or Lynch Syndrome. We hope to address these points in future work. Similarly, we did not assess serial miRNA profiles among the same individuals. Whether the *BRCA*ness profile is static or dynamic requires further study. We also limited our study to female participants. Whether this same signature would be reflected in men with HBOC is unknown. Finally, it is noted that the external validation set is limited to ovarian cancer testing, and we also did not externally validate the model directly for BRCA carrier testing. The PLCO study focuses on ovarian cancer and assesses patients over a 5-year period and is thus appropriate to test the model efficacy when evaluating long-term ovarian cancer risk. We tested the model internally (i.e., using the biobank data and cross-validation) for predicting breast cancer risk, but have not yet evaluated the model on an external set for breast cancer. In further work, we aim to test our model for long-term assessment of breast cancer on a separate cohort of women where the study is designed for long-term monitoring of breast cancer.

In conclusion, we present a highly robust and accurate model for identifying *BRCA* mutation carriers. Based on this model, we also propose a *BRCAness score*, which can be used to inform future cancer risk. The proposed test can be performed at greatly reduced cost and increased efficiency compared to conventional genetic testing. The results appear to be biologically relevant based on the enrichment analysis and could have significant clinical implications for evaluating cancer risk at a population level. The result may improve our ability to identify individuals at risk for HBOC and to implement new cancer prevention and risk management strategies.

## Methods

### Study population

This study including use of samples from the MGB Biobank and PLCO was approved by the Mass General Brigham Institutional Review Board under Protocol #2018P001680. Serum samples from study subjects participating in the Mass General Brigham Biobank enrolled between 2012 and 2022^29^. All study subjects provided written informed consent for participation. All research was performed in accordance with relevant guidelines and regulations and in accordance with the Declaration of Helsinki. Subjects were selected for the current study based on a documented visit with a gynecologist in the electronic health record. Demographic characteristics and medical histories were abstracted from the medical record using manual chart review. Race and ethnicity were self-identified within the medical record, and for purposes of analysis were defined as white non-Hispanic versus all other. Mutation carriers were identified by a documented germline genetic testing report in the electronic health record. Serum samples from the Prostate, Lung, Colorectal, and Ovarian Cancer (PLCO) Screening Trial were collected between 1993 and 2001^30^. All participants provided written informed consent. PLCO comprised a randomized controlled trial of 78,216 women aged 55–74 years old from 10 centers across the United States. Participants in the ovarian screening intervention arm were offered annual screening with CA-125 for 6 years and transvaginal ultrasound for 4 years or usual medical care. Samples were obtained through the National Cancer Institute’s Division of Cancer Prevention through the Etiology and Early Marker Studies (EEMS) program. Cases were selected based on a diagnosis of ovarian cancer within 5 years of the index blood draw. Controls were matched to cases by participant age, year of blood draw, and collection center.

### miRNA profiling

miRNA profiles were generated for 179 different miRNA species using Fireplex® probes (Abcam, Cambridge, MA) and measured in mean fluorescence units (MFI) using Guava Easycyte 5HT flow cytometers (Luminex, Austin, TX) according to the manufacturer’s instructions. The panel of miRNAs was optimized to capture serum miRNAs detectable in at least 50% of samples based on our prior next generation sequencing study^10^. Twenty-five μL of serum were utilized for each sample. As the assay can profile up to 68 miRNAs per well on a 96-well plate, each biologic sample was distributed across three assay panels to construct the full 179 miRNA profile, with some overlap between panels to allow for quality control. Each plate also included a well of pooled human serum, water controls, and spike-in reference miRNAs. The panel includes off-species control probes targeting *c. elegans* miRNAs to establish background signal levels. Samples were processed using a STARlet liquid handling robot (Hamilton Robotics, Franklin, MA) and analyzed using the FirePlex® Analysis Workbench software (http://www.abcam.com/FireflyAnalysisSoftware). Technical replicates were not performed, but the coefficient of variation between individual miRNA values for the same sample averages less than 20%. Any outlier samples in terms of quality control using the off-species miRNAs and reference miRNAs were repeated.

### Lasso-based model

We employ a lasso-based Dimensionality Reduction (DR) approach combined with a linear classification model to classify *BRCA* vs non-*BRCA.* Let $X_{1} \in { \mathbb{R}}^{{n \times p_{1} }}$ be a matrix of normalized miRNA expression values, where *n* is the number of samples and *p*_*1*_ the number of miRNAs, and let $X_{2} \in { \mathbb{R}}^{{n \times p_{2} }}$ be a matrix of metadata, where *p*_2_ is the number of metadata variables. For example, the column of *X*_2_ which corresponds to *BRCA* family history (see Table 1) is a binary vector (i.e., its entries are 0 or 1), where 0 indicates no *BRCA* family history, and 1 indicates *BRCA* family history. The data were normalized to have maximum absolute value one and centered to zero mean, so the normalized miRNA expressions and metadata variables are of the same magnitude. Let $Y \in \left\{ {0,1} \right\}^{n}$ be a binary vector of class labels, where 0 indicates non-*BRCA*, and 1 indicates *BRCA*. To reduce the dimension of the miRNA and metadata, we use a lasso^31^. Specifically, we aim to find

1 $$\mathop {\arg \min }\limits_{{{\mathbf{v}}_{1} \in {\mathbb{R}}p1}} \left\| {X_{1} {\mathbf{v}}_{1} - Y} \right\|_{2}^{2} + \beta_{1} \left\| {{\mathbf{v}}_{1} } \right\|_{1} \quad {\mathrm{and}}\quad \mathop {\arg \min }\limits_{{{\mathbf{v}}_{2} \in {\mathbb{R}}p2}} \left\| {X_{2} {\mathbf{v}}_{2} - Y} \right\|_{2}^{2} + \beta_{2} \left\| {{\mathbf{v}}_{2} } \right\|_{1} ,$$

where ${\mathbf{v}}_{1} = \sum\nolimits_{i} {\left| {v_{i} } \right|}$ denotes *L*^1^ norm, and β_1_; β_2_ > 0 are regularization parameters which control the level of sparsity in **v**_1_; **v**_2._, respectively. The lasso models were fit using the “lasso” Matlab function. Once **v**_1_ and **v**_2_ are determined, the miRNA and metadata are mapped to two-dimensional space:

$$\left[ {X_{1} ,X_{2} } \right] \to X = \left[ {X_{1} {\mathbf{v}}_{1} ,X_{2} {\mathbf{v}}_{2} } \right].$$

Then, to classify subjects as *BRCA* or non-*BRCA*, a linear classification model is trained on *X*

$$P\left( {y = j|{\mathbf{X}}} \right) = \frac{{e^{{{\mathbf{x}}^{T} {\mathbf{w}}_{j} + {\mathbf{b}}_{j} }} }}{{\sum\nolimits_{i = 0}^{{n_{c} - 1}} {e^{{{\mathbf{x}}^{T} {\mathbf{w}}_{i} + {\mathbf{b}}_{i} }} } }},$$

where $j \in \left\{ {0,1} \right\}$ is the class label, ${\mathbf{x}} \in { \mathbb{R}}^{2}$ is a sample in reduced dimension space (i.e., one row of *X*), and the (${\mathbf{w}}_{{\boldsymbol{i}}} , {\mathbf{b}}_{{\boldsymbol{i}}}$) are weights and biases to be trained. Here *y* denotes the class label assigned to x. A subject is then classified as having BRCA mutation if $P\left( {j = 1,x} \right) > t$, where $t \in \left[ {0,1} \right]$ is the BRCA threshold. The classifier was trained using the “trainSoftmaxLayer” Matlab function. To validate the lasso-based model, we use tenfold cross validation. The hyperparameters, β_1_ and β_2_, are chosen using nested tenfold validation on each training fold.

In the main text, a “*BRCA* score” is discussed. The *BRCA* score is defined $b_{s} = {\mathbf{x}}^{T} {\mathbf{w}}_{1} + {\mathbf{b}}_{1}$, which is then translated and scaled to be within the range [0,1], for better interpretability. After the model was trained using all training data, the weights and biases were ${\mathbf{w}}_{1} = \left( {5.6357, 7.4701} \right)^{T}$ and ${\mathbf{b}}_{1} = - 3.1716$, with ${\mathbf{w}}_{2} = - {\mathbf{w}}_{1}$ and ${\mathbf{b}}_{2} = - {\mathbf{b}}_{1}$. See the supplemental tables for the corresponding values of the weights ${\mathbf{v}}_{1}$ and ${\mathbf{v}}_{2}$. To calculate the relative risk curves in Supplementary Fig. S4B and D, and Fig. 6B and D, the *BRCA* score was broken up into bins with width 0.2 and the relative cancer risk was calculated among subjects within that bin. The bins had centers from 0 to 1 in steps of 0.05 (21 bins in total, some of which overlap). To explain in more detail, first, we calculated the baseline risk *R*_*b*_ = (*total cancers in first bin*)/(*total samples in first bin*), where the first bin contains those patients with BRCA score between 0 and 0.1. Then, for each BRCA score bin, e.g., subjects with BRCA score between 0.5 and 0.7, we calculated *R*_*a*_ = (*total cancers in given bin*)/(*total samples in given bin*). For the relative risk curves in Supplementary Fig. S4, the data used in the calculation of $R_{b}$ and $R_{a}$ is the biobank (training) data, and for Fig. 6, we use the PLCO data. The relative risk is calculated as $R_{a}$/$R_{b}$. Once the relative risk was calculated for all 21 bins, we fit a straight line between the BRCA score and the log relative risk, based on the non-linear nature of the observed upward trend, and calculated the correlation.

In Supplementary Fig. S5, we present a schematic of our classification procedure. On the left-hand side, we show 3-D t-distributed Stochastic Neighbor Embedding (TSNE) plots of the miRNA and metadata, in order to visualize the two data sets and show how the *BRCA* and non-*BRCA* subjects separate. The plots indicate a mild, linear separation of the *BRCA* and non-*BRCA* classes. In the center of Fig. S5, we show the result of the lasso-based dimension reduction (i.e., *X*). The miRNA feature ($X_{1} {\mathbf{v}}_{1}$) is shown on the y axis, and the metadata feature ($X_{2} {\mathbf{v}}_{2}$) is on the x axis. There is significant linear separation between *BRCA* and non-*BRCA* subjects in the reduced dimension space. We have split the *X* space into two parts, one the likely *BRCA* group, and the other the likely non-*BRCA* group. Once the miRNA and metadata are projected into 2-D space as in the central scatter plot, the classifier (illustrated on the right-hand of Fig. S5) assigns a probability to *BRCA* (i.e., $P\left( {j = 1,x} \right)$) and non-*BRCA* ($P\left( {j = 0,x} \right)$) as described above. Then, we define a threshold $t \in \left[ {0,1} \right]$ and classify the subject as a *BRCA* mutation carrier if their mutation probability is greater than *t*, and conversely as a non-carrier if the probability of *BRCA* is less than or equal to *t*. The parameter* t* allows us to decide whether to favor model specificity or sensitivity. For example, setting *t* closer to zero gives more weight to sensitivity, and vice-versa.

### Over-representation analysis

A miRNA over-representation analysis was performed using the publicly available miRNA Enrichment Analysis and Annotation Tool (miEAA) from Saarland University^32^. The 20 miRNAs from the limited feature model were entered to examine statistically significant associations with defined categories with Benjamini–Hochberg adjustment for *p* value of < 0.05. Significant target gene associations were entered into STRING v.11.0 to model potential signaling relationships (STRING Consortium 2020, https://string-db.org/).

## Supplementary Information

Below is the link to the electronic supplementary material.

Supplementary Material 1

Supplementary Material 2

Supplementary Material 3

## Data Availability

Raw data files with deidentified metadata and miRNA expression profiles are available in the supplementary materials or at the following link: https://kaggle.com/datasets/a8b2c623d8a7ad1e5c61ec9a61dfc9c3b94fa66870f92ac2e81f4e04684c78f9.
