# Supplementary material for: A lasso-based model combining miRNA and clinical variables predicts future risk of breast and ovarian cancer
Source: Sci Rep. 2026 Mar 24;16:14813. doi: 10.1038/s41598-026-45020-3 (PMC13168662; doi:10.1038/s41598-026-45020-3)
Supplement: Supplementary file 1 — Supplementary Material 1 [file 41598_2026_45020_MOESM1_ESM.rtf]

Ovarian MIRNA: Data Dictionary

Table of Contents

Section Number	Section Title	
1	Study Information	
2	Matching	
3	Predictions	
4	Identifiers	
5	Trial Entry	
6	CDCC Transfer	
7	BQ Compliance	
8	BQ Demographics	
9	BQ Smoking	
10	BQ Family History	
11	BQ Body Type	
12	BQ NSAIDS	
13	BQ Diseases	
14	BQ Female Specific	
15	BQ Screening History	
16	Cancer Diagnosis	
17	Exit	
18	Cancer Characteristics	
19	Treatments	
20	Pathology Images	
21	Screening	
22	Diagnostic Workup	
23	Mortality Status	
24	Death Certificate Cause of Death	
25	Final Cause of Death	
26	BQ Cohort Entry	
27	Other Cohort Entry	
28	Cohort Eligibility	


 
Document Summary


Property	Value	
Document Title	Ovarian MIRNA: Data Dictionary	
Date Created	04/28/2023	
Sections	28	
Entries	218	
Document Filename	dictionary_2017-0023-aug21-d042823.rtf	


 
Ovarian MIRNA: Data Dictionary

Section 1: Study Information


Class	Variable	Label	Description	Format Text	
01. Principal	current_material_yr	Current Material Year for This Obs		numeric	
01. Principal	is_case	Case/Control Status		0="Non-Case"
1="Case"	
01. Principal	patient_id	Patient ID		char 9	


 
Section 2: Matching


Class	Variable	Label	Description	Format Text	
01. Principal	caseset	Case Set		numeric	
01. Principal	match_agelevel	Matched Age Level at Randomization		0="55-59"
1="60-64"
2="65-69"
3="70+"	
01. Principal	match_cig_stat	Matched Smoking Status		0="Never"
1="Former"
2="Current"	
01. Principal	match_fiscal_rndyear	Matched Fiscal Year of Randomization		numeric	
01. Principal	match_material_yr	Selected Material Year		numeric	
01. Principal	match_race3	Matched Race		1="White"
2="Black"
3="Other"	
01. Principal	match_study_yr	Years from Draw to DX/Selection		numeric	


 
Section 3: Predictions


Class	Variable	Label	Description	Format Text	
01. Principal	cancer_probability	Cancer Probability		numeric
.="Missing"	
01. Principal	closest_to_roc_corner	Closest to ROC Corner		numeric
.="Missing"	
01. Principal	per_50	50 Percent		numeric
.="Missing"	
01. Principal	sens_95	95 Sens		numeric
.="Missing"	
01. Principal	sens_99	99 Sens		numeric
.="Missing"	
01. Principal	spec_95	95 Spec		numeric
.="Missing"	
01. Principal	spec_99	99 Spec		numeric
.="Missing"	


 
Section 4: Identifiers


Class	Variable	Label	Description	Format Text	
01. Principal	build	Build	Masterfile build, used to identify the version of the database.	Char, 30	
01. Principal	build_cancers	Build Cancer Cohort	The source of the cancer incidence data.	1="Trial Only"	
01. Principal	build_incidence_cutoff	Build Incidence Cutoff	The censoring cutoff for cancer incidence data associated with the dataset.	0="Uncensored Cancer Incidence Data"	
01. Principal	ovary_trial_flag	Had Ovaries at Baseline	Did the participant have their ovaries at baseline?	0="No"
1="Yes"	
01. Principal	plco_id	PLCO ID	PLCO ID	Char	


 
Section 5: Trial Entry


Class	Variable	Label	Description	Format Text	
01. Principal	age	Age At Randomization	Age at trial entry, computed from date of birth and randomization date.	Numeric	
01. Principal	agelevel	Age At Randomization	Categorical version of age, created from the derived age variable.	0="<= 59"
1="60-64"
2="65-69"
3=">= 70"	
01. Principal	arm	Randomization Arm	Randomization group or arm.
The intervention (screening) group or the control (usual-care) group.	1="Intervention"	
01. Principal	center	Study Center	The study center at which the participant was randomized.	1="University of Colorado"
2="Georgetown University"
3="Pacific Health Research and Education Institute (Honolulu)"
4="Henry Ford Health System"
5="University of Minnesota"
6="Washington University in St Louis"
8="University of Pittsburgh"
9="University of Utah"
10="Marshfield Clinic Research Foundation"
11="University of Alabama at Birmingham"	
01. Principal	ph_any_trial	Trial Personal History of Any Cancer	Did the participant have a personal history of any cancer prior to trial entry?	0="No"
1="Yes"	
01. Principal	rndyear	Year Of Randomization	Calendar year of trial entry, at which point the participant was randomized into an arm.	Numeric	
01. Principal	sex	Sex	Sex of the participant.	2="Female"	
05. Permutations	dual	Screening Dual Consent	Was the participant presented with single consent or dual consent? Three study centers (Henry Ford Health System, Washington University in St Louis and Pacific Health Research Institute in Honolulu) chose the dual consent approach initially. All three centers switched to single consent from 1995 to 1997. 

	0="No, single consent"
1="Yes, dual consent"	
05. Permutations	ph_ovar_trial	Trial Personal History of Ovarian/Ft/Pt Cancer	Did the participant have a personal history of ovarian/ft/pt cancer prior to trial entry?	0="No"	


 
Section 6: CDCC Transfer


Class	Variable	Label	Description	Format Text	
01. Principal	reconsent_outcome	Outcome of the Re-Consent Process	The immediate result of the attempt to transfer the participant to centralized follow-up.

Participants alive at trial close-out were informed of the transfer to centralized follow-up, and could request/refuse continued follow-up.  In the absence of participant contact, the re-consent outcome was determined by default.  The default outcome varied by study center.
Active: Transferred to centralized follow-up, including continued contact, state registry query and NDI search.
Passive: No active contact, but allows for state registry query and NDI search.
Lost: Lost to follow-up and not known dead before re-consent. Considered passive at the time of reconsent.
Refused: Declined further follow-up.  
Confirmed/Presumed Dead: Death prior to opportunity to re-consent.	1="Active by Request"
2="Active by Default/Follow-up"
3="Passive by Request"
4="Passive by Default/Follow-up"
5="Refused by Request"
6="Refused by Default/Follow-up"
11="Refused Prior to Re-Consent"
12="Confirmed Dead Prior to Re-Consent"
14="Passive, Lost Prior to Re-Consent"	
01. Principal	reconsent_outcome_days	Days Until Re-Consent Outcome is Determined	Days from randomization until the participant's re-consent outcome is determined.

For participants who re-consented by request or refused by request, this is the time of request.  For participants whose transfer status was determined by default, this is an estimated time of request had there been contact.  If the participant had refused or died prior to re-consent, this is the time of those events.	Numeric	


 
Section 7: BQ Compliance


Class	Variable	Label	Description	Format Text	
01. Principal	bq_adminm	Method of Questionnaire Administration	Part of the section, For Office Use Only, headed "Method of Administration".	.M="Not Answered"
1="Self"
2="Self With Assistance"
3="In-Person Interview By SC Staff"
4="In-Person Interview By Other"
5="Telephone"	
01. Principal	bq_age	Age at BQ	Calculated from date of baseline questionnaire completion and date of birth.	Numeric	
01. Principal	bq_compdays	Days Until BQ Completion	Question M48, F63 - "What is the date you completed this questionnaire?"

The number of days between BQ completion and randomization.	Numeric	
01. Principal	bq_returned	Did the Participant Return the BQ?		1="Yes"	


 
Section 8: BQ Demographics


Class	Variable	Label	Description	Format Text	
01. Principal	educat	Education	Question 3 - "What is the highest grade or level of schooling you completed?"	1="Less Than 8 Years"
2="8-11 Years"
3="12 Years Or Completed High School"
4="Post High School Training Other Than College"
5="Some College"
6="College Graduate"
7="Postgraduate"	
01. Principal	hispanic_f	Are You Of Hispanic Origin?	BQ Form Versions 1 and 2: Question 2. BQ Form Version 3: Question 2a. 

What is your race or ethnicity?	.M="Not Answered"
0="Not Hispanic"
1="Hispanic"	
01. Principal	marital	Marital Status	Question 4 - "What is your current marital status?"	.M="Not Answered"
1="Married Or Living As Married"
2="Widowed"
3="Divorced"
4="Separated"
5="Never Married"	
01. Principal	occupat	Occupation	Question 5 - "Which of these categories best describes your current working situation?"	.M="Not Answered"
1="Homemaker"
2="Working"
3="Unemployed"
4="Retired"
6="Disabled"
7="Other"	
01. Principal	race7	Race	BQ Form Versions 1 and 2: Question 2 - "Which of these best describes your race or ethnic background?"

BQ Form Version 3: Question 2 - "Which of these groups best describes you?" Question 2a - "Are you of Hispanic origin?"

Participants can only be considered white or black when they are not Hispanic. If the participant is white or black and Hispanic, then they are considered Hispanic. If the participant is Asian, Pacific Islander, or American Indian then they are considered that race.	1="White, Non-Hispanic"
2="Black, Non-Hispanic"
3="Hispanic"
4="Asian"
6="American Indian"	


 
Section 9: BQ Smoking


Class	Variable	Label	Description	Format Text	
01. Principal	cig_stat	Cigarette Smoking Status	Participant's current cigarette smoking status.	0="Never Smoked Cigarettes"
1="Current Cigarette Smoker"
2="Former Cigarette Smoker"	
01. Principal	cig_stop	# of Years Since Stopped Smoking Cigarettes	The number of years passed since the participant has stopped smoking.	Numeric
.M="Not Answered"
.N="Not Applicable"
0.5="Six Months"	
01. Principal	cig_years	Duration Smoked Cigarettes	The total number of years the participant smoked.	Numeric
.M="Not Answered"	
01. Principal	cigar	Ever Smoked Cigars?	Question 17 - "Do you now or did you ever smoke cigars regularly for a year or longer?"	.M="Not Answered"
0="Never"
1="Current Cigar Smoker"
2="Former Cigar Smoker"	
01. Principal	cigpd_f	# of Cigarettes Smoked Per Day	Question 14 - "During periods when you smoked, how many cigarettes did or do you usually smoke per day?"	0="0"
1="1-10"
2="11-20"
3="21-30"
4="31-40"
5="41-60"
6="61-80"	
01. Principal	filtered_f	Usually Filtered or Non-Filtered?	Question 15 - "During periods when you smoked, did or do you more often smoke filter or non-filter cigarettes?"	.N="Not Applicable"
1="Filter"
2="Non-Filter"
3="About Equal"	
01. Principal	pack_years	Pack Years	Number of packs smoked per day * years smoked.	Numeric
.M="Missing"	
01. Principal	pipe	Ever Smoked a Pipe?	Question 16 - "Do you now or did you ever smoke a pipe regularly for a year or longer?"	.M="Not Answered"
0="Never"
2="Former Pipe Smoker"	
03. Components	rsmoker_f	Smoke Regularly Now?	Question 12 - "Do you smoke cigarettes regularly now?"	.N="Not Applicable"
0="No"
1="Yes"	
03. Components	smokea_f	Age Started Smoking	Question 11 - "At what age did you start smoking cigarettes regularly?"	Numeric
.M="Not Answered Or Inconsistent Data"
.N="Not Applicable"	
03. Components	smoked_f	Ever Smoke Regularly >= 6 Months?	Question 10 - "Have you ever smoked cigarettes regularly for six months or longer?"	0="No"
1="Yes"	
03. Components	ssmokea_f	Age Stopped Smoking	Question 13 - "At what age did you last stop smoking cigarettes regularly?"	Numeric
.M="Not Answered Or Inconsistent Data"
.N="Not Applicable"
.R="Age not in reasonable range."	


 
Section 10: BQ Family History


Class	Variable	Label	Description	Format Text	
01. Principal	brothers	# of Brothers	Question 19 - "How many full and half-brothers do you have, both living and deceased?"

Participants who have more than seven brothers are collapsed into "7 or more."	.M="Not Answered"
0="None"
1="One"
2="Two"
3="Three"
4="Four"
5="Five"
6="Six"
7="Seven Or More"	
01. Principal	fh_cancer	Has Family History of Any Cancer?	Any first-degree relative with cancer. Basal cell skin cancers are not included. First-degree relatives include parents, full-siblings, and children. Half-siblings are not included.	.M="Not Answered"
0="No"
1="Yes"	
01. Principal	sisters	# of Sisters	Question 18 - "How many full and half-sisters do you have, both living and deceased?"

Participants with more than seven sisters are collapsed into "7 or more".	.M="Not Answered"
0="None"
1="One"
2="Two"
3="Three"
4="Four"
5="Five"
6="Six"
7="Seven Or More"	
05. Permutations	breast_fh	Family History of Female Breast Cancer	Breast cancer family history in first-degree relatives. Includes parents, full-siblings, and children.	.M="Missing"
0="No"
1="Yes, Immediate Female Family Member"
2="Male Relative Only"
9="Possibly - Relative Or Cancer Type Not Clear"	
05. Permutations	breast_fh_age	Age of Youngest Relative with Breast Cancer	Diagnosis age of the youngest first-degree relative diagnosed with breast cancer.	Numeric
.A="Ambiguous"
.M="Missing"
.N="Not Applicable"	
05. Permutations	breast_fh_cnt	# of Relatives with Breast Cancer	The number of first-degree relatives with breast cancer.	Numeric
.M="Missing"	
05. Permutations	ovarsumm_fh	Family History of Ovarian Summary Cancer	Ovarian, fallopian tube, or peritoneal cancers family history in first-degree relatives. Includes parents, full-siblings, and children. Ovarian summary cancers include ovarian, fallopian tube and peritoneal cancers.	.M="Missing"
0="No"
1="Yes, Immediate Family Member"
9="Possibly - Relative Or Cancer Type Not Clear"	
05. Permutations	ovarsumm_fh_age	Age of Youngest Relative with Ovarian Summary Cancer	Diagnosis age of the youngest first-degree relative diagnosed with ovarian, fallopian tube, or peritoneal cancer. Ovarian summary cancers include ovarian, fallopian tube and peritoneal cancers.	Numeric
.M="Missing"
.N="Not Applicable"	
05. Permutations	ovarsumm_fh_cnt	# of Relatives with Ovarian Summary Cancer	The number of first-degree relatives with ovarian, fallopian tube, or peritoneal cancer. Ovarian summary cancers include ovarian, fallopian tube and peritoneal cancers.	Numeric
.M="Missing"	


 
Section 11: BQ Body Type


Class	Variable	Label	Description	Format Text	
01. Principal	bmi_20	BMI at Age 20	BMI is considered out of range if any of the following occur:
- Weight is less than 60 pounds
- Height is less than 48 inches
- Height is greater than 78 inches for females
- Height is greater than 84 inches for males
- After BMI is calculated, BMI is less than 15	Numeric
.M="Not Answered"	
01. Principal	bmi_20c	BMI at Age 20	This is the World Health Organization (WHO) standard categorization of BMI. 
BMI is considered out of range if any of the following occur:
- Weight is less than 60 pounds
- Height is less than 48 inches
- Height is greater than 78 inches for females
- Height is greater than 84 inches for males
- After BMI is calculated, BMI is less than 15	.M="Not Answered"
1="0-18.5"
2="18.5-25"
3="25-30"
4="30+"	
01. Principal	bmi_50	BMI at Age 50	BMI is considered out of range if any of the following occur:
- Weight is less than 60 pounds
- Height is less than 48 inches
- Height is greater than 78 inches for females
- Height is greater than 84 inches for males
- After BMI is calculated, BMI is less than 15	Numeric
.M="Not Answered"	
01. Principal	bmi_50c	BMI at Age 50	This is the World Health Organization (WHO) standard categorization of BMI. 
BMI is considered out of range if any of the following occur:
- Weight is less than 60 pounds
- Height is less than 48 inches
- Height is greater than 78 inches for females
- Height is greater than 84 inches for males
- After BMI is calculated, BMI is less than 15	.M="Not Answered"
1="0-18.5"
2="18.5-25"
3="25-30"
4="30+"	
01. Principal	bmi_curc	BMI at Baseline	This is the World Health Organization (WHO) standard categorization of BMI. 
BMI is considered out of range if any of the following occur:
- Weight is less than 60 pounds
- Height is less than 48 inches
- Height is greater than 78 inches for females
- Height is greater than 84 inches for males
- After BMI is calculated, BMI is less than 15	.M="Not Answered"
1="0-18.5"
2="18.5-25"
3="25-30"
4="30+"	
01. Principal	bmi_curr	BMI at Baseline	BMI is considered out of range if any of the following occur:
- Weight is less than 60 pounds
- Height is less than 48 inches
- Height is greater than 78 inches for females
- Height is greater than 84 inches for males
- After BMI is calculated, BMI is less than 15	Numeric
.M="Not Answered"	
01. Principal	height_f	Height (inches)	Question 23 - "How tall are you?" 

Height is considered out of range if any of the following occur:
- Height is less than 48 inches
- Height is greater than 78 inches for females
- Height is greater than 84 inches for males
- After BMI is calculated, BMI is less than 15	Numeric
.M="Missing"	
01. Principal	weight20_f	Weight at Age 20 (lbs)	Question 22 - "What is or was your weight at these ages?" 

Weights less than 60 pounds are out of range.	Numeric
.M="Missing"	
01. Principal	weight50_f	Weight at Age 50 (lbs)	Question 22 - "What is or was your weight at these ages?" 

Weights less than 60 pounds are out of range.	Numeric
.M="Missing"	
01. Principal	weight_f	Weight (lbs) at Baseline	Question 22 - "What is or was your weight at these ages?" 

Weights less than 60 pounds are out of range.	Numeric
.M="Missing"	


 
Section 12: BQ NSAIDS


Class	Variable	Label	Description	Format Text	
01. Principal	asp	Use Aspirin Regularly?	Question 24 - "During the last 12 months, have you regularly used aspirin or aspirin-containing products, such as Bayer, Bufferin or Anacin? (Please do not include aspirin-free products such as Tylenol and Panadol.)"	0="No"
1="Yes"	
01. Principal	asppd	# of Aspirin	Question 25 - "During the last 12 months, how many pills of aspirin or aspirin containing products did you usually take per day, per week or per month?"	.M="Not Answered"
0="None"
1="1/Day"
2="2+/Day"
3="1/Week"
4="2/Week"
5="3-4/Week"
6="<2/Month"
7="2-3/Month"	
01. Principal	ibup	Use Ibuprofen Regularly?	Question 26 - "During the last 12 months, have you regularly used ibuprofen-containing products, such as Advil, Nuprin, or Motrin?"	0="No"
1="Yes"	
01. Principal	ibuppd	# of Ibuprofen	Question 27 - "During the last 12 months, how many pills of ibuprofen-containing products did you usually take per day, per week, or per month?"	.M="Not Answered"
0="None"
1="1/Day"
2="2+/Day"
3="1/Week"
4="2/Week"
5="3-4/Week"
6="<2/Month"
7="2-3/Month"	


 
Section 13: BQ Diseases


Class	Variable	Label	Description	Format Text	
01. Principal	arthrit_f	Arthritis	Did the participant ever have arthritis?	.M="Not Answered"
0="No"
1="Yes"	
01. Principal	bronchit_f	Bronchitis	Did the participant ever have chronic bronchitis?	0="No"
1="Yes"	
01. Principal	colon_comorbidity	Colon Comorbidities	Did the participant ever have a colon related co-morbidity (ulcerative colitis, Crohn's disease, Gardner's syndrome, or familial polyposis)?	.M="Missing"
0="No"
1="Yes"	
01. Principal	diabetes_f	Diabetes	Did the participant ever have diabetes?	0="No"
1="Yes"	
01. Principal	divertic_f	Diverticulitis/Diverticulosis	Did the participant ever have diverticulitis or diverticulosis?	.M="Not Answered"
0="No"
1="Yes"	
01. Principal	emphys_f	Emphysema	Did the participant ever have emphysema?	0="No"
1="Yes"	
01. Principal	gallblad_f	Gallbladder Stones or Inflammation	Did the participant ever have gall bladder stones or inflammation?	.M="Not Answered"
0="No"
1="Yes"	
01. Principal	hearta_f	Heart Attack	Did the participant ever have coronary heart disease or a heart attack?	0="No"
1="Yes"	
01. Principal	hyperten_f	Hypertension	Did the participant ever have high blood pressure?	0="No"
1="Yes"	
01. Principal	liver_comorbidity	Liver Comorbidities	Did the participant ever have a liver related co-morbidity (hepatitis or cirrhosis)?	.M="Missing"
0="No"
1="Yes"	
01. Principal	osteopor_f	Osteoporosis	Did the participant ever have osteoporosis?	.M="Not Answered"
0="No"
1="Yes"	
01. Principal	polyps_f	Colorectal Polyps	Did the participant ever have colorectal polyps?	.M="Not Answered"
0="No"
1="Yes"	
01. Principal	stroke_f	Stroke	Did the participant have a stroke?	0="No"
1="Yes"	


 
Section 14: BQ Female Specific


Class	Variable	Label	Description	Format Text	
01. Principal	bbd	Ever Have Benign or Fibrocystic Breast Disease?	Question F54 - "Have you ever been told by a doctor that you had any of the following conditions?"	.M="Not Answered"
0="No"
1="Yes"	
01. Principal	bcontr_f	Ever Take Birth Control Pills?	Question F43 - "Did you ever take birth control pills for birth control or to regulate menstrual periods?".

Participant's answer modified to "yes" if they specified both an age they started taking birth control pills and a total number of years they took them.	0="No"
1="Yes"	
01. Principal	bcontra	Age Started Birth Control Pills?	Question F44 - "How old were you when you first started taking birth control pills?"

Participants who were "50-59" or "60+" when they started birth control pills were collapsed into a "50+" category.	.M="Not Answered"
.N="Not Applicable"
1="<30"
2="30-39"
3="40-49"
4="50+"	
01. Principal	bcontrt	Total Years Took Birth Control Pills?	Question F45 - "For how many total years did you take birth control pills?"	.M="Not Answered"
0="Not Applicable"
1="10+ Years"
2="6-9 Years"
3="4-5 Years"
4="2-3 Years"
5="1 Year or Less"	
01. Principal	benign_ovcyst	Ever Have Benign Ovarian Tumor/Cyst?	Question F54 - "Have you ever been told by a doctor that you had any of the following conditions?"	.M="Not Answered"
0="No"
1="Yes"	
01. Principal	endometriosis	Ever Have Endometriosis?	Question F54 - "Have you ever been told by a doctor that you had any of the following conditions?"	.M="Not Answered"
0="No"
1="Yes"	
01. Principal	fchilda	Age at Birth of First Child?	Question F42 - "What was your age at the birth of your first child?"	.M="Not Answered"
.N="Not Applicable"
1="<16"
2="16-19"
3="20-24"
4="25-29"
5="30-34"
6="35-39"
7="40+"	
01. Principal	fmenstr	Age When Had First Menstrual Period?	Question F31 - "How old were you when you had your first menstrual period?"	.M="Not Answered"
1="<10"
2="10-11"
3="12-13"
4="14-15"
5="16+"	
01. Principal	horm_stat	Female Hormone Status	Female hormone status uses ever taken female hormones and currently on hormones to determine the participant's hormone status.	.M="Missing"
0="Never"
1="Current"
2="Former"
4="Doesn't Know If She Ever Took HRT"	
01. Principal	hyster_f	Ever Have a Hysterectomy?	Question F47 - "Have you had a hysterectomy, that is, have you had your uterus or womb removed?"

Participants modified to "yes" if an age of hysterectomy is given in question F48	0="No"
1="Yes"
2="Don't Know"	
01. Principal	hystera	Age at Hysterectomy	Question F48 - "What was your age when you had your uterus or womb removed?"	.M="Not Answered"
.N="Not Applicable"
1="<40"
2="40-44"
3="45-49"
4="50-54"
5="55+"	
01. Principal	livec	# of Live Births	Question F41 - "How many of your pregnancies resulted in a live birth?"

Allowed values are 0-29. Participants with more than five pregnancies are collapsed to "five or more".	.M="Not Answered"
0="Zero"
1="One"
2="Two"
3="Three"
4="Four"
5="Five Or More"	
01. Principal	lmenstr	Age at Menopause	Question F32 - "How old were you when you had your last period?"	.M="Not Answered"
1="<40"
2="40-44"
3="45-49"
4="50-54"
5="55+"	
01. Principal	menstrs	Type of Menopause	Question F33 - "Did your periods stop because of natural menopause, surgery, radiation, or drug therapy?"	.M="Not Answered"
1="Natural Menopause"
2="Surgery"
3="Radiation"
4="Drug Therapy"	
01. Principal	menstrs_stat_type	Reason menstrual periods stopped.	Reason the participant's menstrual periods stopped. Because minimal information was gathered about menopause, the menopause information is supplemented with hysterectomy and oophorectomy information.	1="Natural postmenopausal"
2="Bilateral oophorectomy"
3="Hysterectomy, no bilateral oophorectomy"
4="Surgical, details unclear"
5="Drug therapy"
6="Radiation"
8="Menopausal status unknown"	
01. Principal	miscar	# of Miscarriages/Abortions	Question F39 - "How many of your pregnancies resulted in miscarriage or an abortion?"	.M="Not Answered"
0="0"
1="1"
2="2+"	
01. Principal	ovariesr_f	Removed Ovaries	Question F49 - "Have you ever had one or both of your ovaries removed?"

Question F50 - "What exactly was removed?"	.M="Not Answered"
0="Ovaries Not Removed"
1="One Ovary - Partial"
2="One Ovary - Total"
3="Both Ovaries - Partial"
4="Both Ovaries - Total"
5="Don't Know"	
01. Principal	post_menopausal	Post-Menopausal Status	Was the participant post-menopausal at trial entry. This question was not asked directly on the BQ, therefore information on menopause has been supplemented with hysterectomy and oophorectomy information.	1="Definitely post-menopausal"
2="Possibly post-menopausal"	
01. Principal	preg_f	Ever Been Pregnant?	Question F35 - "Have you ever been pregnant?"

Participant's answer is modified to be "yes" if the participant answers on age of first pregnancy, number of pregnancies, number of still birth pregnancies, number of miscarriages, number of tubal pregnancies, age at birth of first child, or the number of live births implied pregnancy.	0="No"
1="Yes"
2="Don't Know"	
01. Principal	prega	Age When First Became Pregnant?	Question F36 - "How old were you when you first became pregnant?"

Participants who were "40-44" or "45+" when they first became pregnant were collapsed into "40+".	.M="Not Answered"
.N="Not Applicable"
1="<15"
2="15-19"
3="20-24"
4="25-29"
5="30-34"
6="35-39"
7="40+"	
01. Principal	pregc	# of Pregnancies	Question F37 - "How many times have you been pregnant? Please include stillbirths, miscarriages, abortions, tubal or ectopic pregnancies, and live births."	.M="Not Answered"
0="None"
1="1"
2="2"
3="3-4"
4="5-9"
5="10+"	
01. Principal	stillb	# of Still Birth Pregnancies	Question F38 - "How many of your pregnancies resulted in a stillbirth?"	.M="Not Answered"
0="0"
1="1"
2="2+"	
01. Principal	thorm	# of Years Taking Female Hormones	Question F53 - "For how many total years did you take female hormones?"	.M="Not Answered"
0="Not Applicable"
1="10+ Years"
2="6-9 Years"
3="4-5 Years"
4="2-3 Years"
5="<= 1 Year"	
01. Principal	trypreg	Ever Tried to Become Pregnant for a Year or More Without Success?	Question F34 - "Have you ever tried to become pregnant for a year or more without success?"	.M="Not Answered"
0="No"
1="Yes"	
01. Principal	tubal	# of Tubal/Ectopic Pregnancies?	Question F40 - "How many of your pregnancies resulted in a pregnancy in one of your tubes, that is, a tubal or ectopic pregnancy?"	.M="Not Answered"
0="0"
1="1"
2="2+"	
01. Principal	tuballig	Ever Tubes Tied?	Question F46 - "Have you had a tubal ligation, that is have you had your tubes tied?"	0="No"
1="Yes"	
01. Principal	uterine_fib	Ever Have Uterine Fibroid Tumors?	Question F54 - "Have you ever been told by a doctor that you had any of the following conditions?"	.M="Not Answered"
0="No"
1="Yes"	
03. Components	curhorm	Currently Using Female Hormones?	Question F52 - "Are you currently using female hormones?"	.M="Not Answered"
0="No"
1="Yes"	
03. Components	horm_f	Ever Take Female Hormones?	Question F51 - "Sometimes women take female hormones such as estrogen or progesterone around the time of menopause.  Have you ever used female hormones (tablets, pills, or creams) for menopause?"

Participant's answers modified to "yes" if they had said "no" but gave an answer for whether they are currently using female hormones and said they used them for greater than 1 year.	.M="Not Answered"
0="No"
1="Yes"
2="Don't Know"	


 
Section 15: BQ Screening History


Class	Variable	Label	Description	Format Text	
05. Permutations	ca125_history	Had Blood Test For Ovarian Cancer in Past 3 Years?	Question F60 - "During the past three years, have you had a blood test for ovarian cancer, for example CA-125?"	0="No"
1="Yes, Once"
2="Yes, More Than Once"
3="Don't Know"	
05. Permutations	mammo_history	Had Mammogram in Past 3 Years?	Question F56 - During the past three years, have you had a mammogram?"	0="No"
1="Yes, Once"
2="Yes. More Than Once"
3="Don't Know"	
05. Permutations	papsmear_history	Had Pap Smear in Past 3 Years?	Question F57 - "During the past three years, have you had a pap smear?"	0="No"
1="Yes, Once"
2="Yes. More Than Once"
3="Don't Know"	
05. Permutations	pelvic_history	Had Pelvic Exam in Past 3 Years?	Question F58 - "During the past three years, have you had a pelvic examination?"	0="No"
1="Yes, Once"
2="Yes. More Than Once"
3="Don't Know"	
05. Permutations	usound_history	Had Ultrasound/Scan of the Ovaries In Past 3 Years?	Question F59 - "During the past three years, have you had an ultrasound or scan of your ovaries?"	0="No"
1="Yes, Once"
2="Yes, More Than Once"
3="Don't Know"	


 
Section 16: Cancer Diagnosis


Class	Variable	Label	Description	Format Text	
01. Principal	ovar_cancer	Confirmed Ovarian Cancer	Does the participant have confirmed primary ovarian, peritoneal, or fallopian tube cancer diagnosed during the trial?	0="No Confirmed Cancer"
1="Confirmed Cancer"	
01. Principal	ovar_cancer_diagdays	Ovarian Confirmed Cancer Diagnosis Days		Numeric
.N="Not Applicable"	
01. Principal	ovar_cancer_first	Was Ovarian Cancer The First Diagnosed Cancer?	Among all of a participant's cancers diagnosed during the trial, was ovarian, peritoneal, or fallopian tube cancer the earliest?	.N="Not Applicable"
0="No"
1="Yes"	
01. Principal	ovar_cancer_site	Ovarian Cancer Type	What type of ovarian cancer was diagnosed?

The ovarian effort includes (true) ovarian, peritoneal, and fallopian tube cancers.	.N="Not Applicable"
1="Invasive Ovarian"
2="Primary Peritoneal"
3="Fallopian Tube"
4="Ovarian Low Malignancy Potential Tumor"
6="Fallopian Tube Low Malignancy Potential Tumor"	
01. Principal	ovar_intstat_cat	Ovarian Cancer Screen Detected vs. Interval Status	Classifies ovarian, peritoneal, and fallopian tube cancers based on their screen detected or interval status.	.N="Not Applicable"
2="Never Screened"
3="Post-Screening"
4="Interval"
5="Screen Dx"	
03. Components	ovar_reasfoll	Reason For Initial Clinical Assessment: Follow-up Of Positive Screen (Cancer Year)?	DEO2-A.5
DEO3-A.2
Was follow-up to a positive screen listed as a reason for the initial visit for clinical assessment in the cancer year?  This question was not asked on version 1 forms.	.F="No Form"
.N="Not Applicable"
0="No"
1="Yes"	
03. Components	ovar_reasoth	Reason For Initial Clinical Assessment: Other (Specify) (Cancer Year)?	DEO2-A.5
DEO3-A.2
Was an other reason listed as a reason for the initial visit for clinical assessment in the cancer year?  This question was not asked on version 1 forms.	.F="No Form"
.N="Not Applicable"
0="No"
1="Yes"	
03. Components	ovar_reassurv	Reason For Initial Clinical Assessment: Surveillance (Cancer Year)?	DEO2-A.5
Was surveillance listed as a reason for the initial visit for clinical assessment in the cancer year?  This question was not asked on version 1 forms, and this answer was not a choice on version 3 forms.	.F="No Form"
.N="Not Applicable"
.V="Not Asked - Version 1 or 3 Form"
0="No"	
03. Components	ovar_reassymp	Reason For Initial Clinical Assessment: Symptomatic (Cancer Year)?	DEO2-A.5
DEO3-A.2
Was being symptomatic listed as a reason for the initial visit for clinical assessment in the cancer year?  This question was not asked on version 1 forms.	.F="No Form"
.N="Not Applicable"
0="No"
1="Yes"	
05. Permutations	ovar_annyr	Ovarian Cancer Anniversary Year	Full years elapsed before ovarian cancer diagnosis (from randomization/trial entry).	Numeric
.N="Not Applicable"	


 
Section 17: Exit


Class	Variable	Label	Description	Format Text	
01. Principal	fstcan_exitage	First Cancer Incidence Exit Age	Age of participant at exit for first cancer incidence. This is age at diagnosis for participants with cancer and age at trial exit otherwise.

Only cancers occuring during the trial are used to determine exit.	Numeric	
01. Principal	fstcan_exitdays	Days Until First Cancer Incidence Exit	Days from trial entry (randomization) to first cancer diagnosis for participants with cancer, or to trial exit otherwise.

Only cancers occuring during the trial are used to determine exit.	Numeric	
01. Principal	fstcan_exitstat	First Cancer Incidence Exit Status	Status of the participant at exit for first cancer incidence.

Only cancers occuring during the trial are used to determine exit.	1="Confirmed Cancer"
3="Last Participant Contact Prior to Unconfirmed Report"
4="Last Participant Contact"
5="Death"
6="Date Lost, Prior to Death"
9="Post-2009 Death, Exit At 12/31/09"	
01. Principal	material_to_fstcan_exitdays	Days from Material Year for This Obs to First Cancer Dx/Exit	Days from material collection to first cancer diagnosis for participants with cancer, or to trial exit otherwise.

Only cancers occuring during the trial are used to determine exit.	numeric	
01. Principal	material_to_ovar_exitdays	Days from Material Year for This Obs to Ovarian Cancer Dx/Exit	Days from material collection to cancer diagnosis for participants with ovarian, peritoneal, or fallopian tube cancer, or to trial exit otherwise.	numeric	
01. Principal	ovar_exitage	Ovarian Incidence Exit Age	Age of participant at exit for ovarian incidence. This is age at diagnosis for participants with ovarian, peritoneal, or fallopian tube cancer and age at trial exit otherwise.	Numeric	
01. Principal	ovar_exitdays	Days Until Ovarian Incidence Exit	Days from trial entry (randomization) to cancer diagnosis for participants with ovarian, peritoneal, or fallopian tube cancer, or to trial exit otherwise.	Numeric	
01. Principal	ovar_exitstat	Ovarian Incidence Exit Status	Status of the participant at exit for ovarian cancer incidence.	1="Confirmed Cancer"
4="Last Participant Contact"
5="Death"
6="Date Lost, Prior to Death"
9="Post-2009 Death, Exit At 12/31/09"	


 
Section 18: Cancer Characteristics


Class	Variable	Label	Description	Format Text	
01. Principal	ovar_behavior	Ovarian Cancer Behavior (ICD-O-2)	DEO1-C.13
DEO2-C.15
DEO3-C.11

ICD-O-2 behavior code.	.N="Not Applicable"
1="Uncertain, Borderline, or Low Malignancy Potential"
3="Malignant"	
01. Principal	ovar_clinstage	Ovarian Clinical Stage (AJCC 5th Edition)	Clinical stage for ovarian, peritoneal, or fallopian tube cancer.	.M="Missing"
.N="Not Applicable"
110="Stage IA"
300="Stage III"
310="Stage IIIA"
320="Stage IIIB"
330="Stage IIIC"
400="Stage IV"	
01. Principal	ovar_clinstage_7e	Ovarian Clinical Stage (AJCC 7th Edition)	Clinical stage for ovarian, peritoneal, or fallopian tube cancer calculated using the AJCC 7th edition staging manual.

The trial collected stage using the AJCC 5th edition staging manual.  The 7th edition stage has been calculated by using the TNM components recorded for the 5th edition stage, but applying the staging algorithm from the 7th edition staging manual.	.M="Missing"
.N="Not Applicable"
110="Stage IA"
300="Stage III"
310="Stage IIIA"
320="Stage IIIB"
330="Stage IIIC"
400="Stage IV"	
01. Principal	ovar_clinstage_m	Ovarian Clinical Stage M Component	DEO2-19a
DEO3-15a	.M="Missing"
.N="Not Applicable"
0="M0"
100="M1"
999="MX"	
01. Principal	ovar_clinstage_n	Ovarian Clinical Stage N Component	DEO2-19a
DEO3-15a	.M="Missing"
.N="Not Applicable"
0="N0"
100="N1"
999="NX"	
01. Principal	ovar_clinstage_t	Ovarian Clinical Stage T Component	DEO2-19a
DEO3-15a	.M="Missing"
.N="Not Applicable"
110="T1a"
120="T1b"
300="T3"
310="T3a"
320="T3b"
330="T3c"
999="TX"	
01. Principal	ovar_grade	Ovarian Cancer Grade (ICD-O-2)	DEO1-C.13
DEO2-C.15
DEO3-C.11

ICD-O-2 grade.	.N="Not Applicable"
1="Well Differentiated; Grade I"
2="Moderately Differentiated; Grade II"
3="Poorly Differentiated; Grade III"
4="Undifferentiated; Grade IV"
5="T Cell; T Precursor"
9="Unknown"	
01. Principal	ovar_histtype	Ovarian Cancer Histopathologic Type		.N="Not Applicable"
1="Serous Cystadenoma"
2="Serous Cystadenocarcinoma"
3="Mucinous Cystadenoma"
4="Mucinous Cystadenocarcinoma"
6="Endometrioid Adenocarcinoma"
8="Clear Cell Cystadenocarcinoma"
31="Adenocarcinoma, NOS/Carcinoma, NOS"
34="Granulosa Cell Tumor, Malignant"
39="Carcinosarcoma/Sarcoma"	
01. Principal	ovar_morphology	Ovarian Cancer Morphology (ICD-O-2)	DEO1-C.13
DEO2-C.15
DEO3-C.11

ICD-O-2 morphology.  Valid range is 8000-9989.	See ICD-O-2 documentation
.N="Not Applicable"	
01. Principal	ovar_pathstage	Ovarian Pathologic Stage (AJCC 5th Edition)	Pathologic stage for ovarian, peritoneal, or fallopian tube cancer.	.M="Missing"
.N="Not Applicable"
100="Stage I"
110="Stage IA"
120="Stage IB"
130="Stage IC"
200="Stage II"
210="Stage IIA"
220="Stage IIB"
230="Stage IIC"
310="Stage IIIA"
320="Stage IIIB"
330="Stage IIIC"
400="Stage IV"	
01. Principal	ovar_pathstage_7e	Ovarian Pathologic Stage (AJCC 7th Edition)	Pathologic stage for ovarian, peritoneal, or fallopian tube cancer calculated using the AJCC 7th edition staging manual.

The trial collected stage using the AJCC 5th edition staging manual.  The 7th edition stage has been calculated by using the TNM components recorded for the 5th edition stage, but applying the staging algorithm from the 7th edition staging manual.	.M="Missing"
.N="Not Applicable"
100="Stage I"
110="Stage IA"
120="Stage IB"
130="Stage IC"
200="Stage II"
210="Stage IIA"
220="Stage IIB"
230="Stage IIC"
300="Stage III"
310="Stage IIIA"
320="Stage IIIB"
330="Stage IIIC"
400="Stage IV"	
01. Principal	ovar_pathstage_m	Ovarian Pathologic Stage M Component	DEO2-19b
DEO3-15b	.M="Missing"
.N="Not Applicable"
0="M0"
100="M1"
999="MX"	
01. Principal	ovar_pathstage_n	Ovarian Pathologic Stage N Component	DEO2-19b
DEO3-15b	.M="Missing"
.N="Not Applicable"
0="N0"
100="N1"
999="NX"	
01. Principal	ovar_pathstage_t	Ovarian Pathologic Stage T Component	DEO2-19b
DEO3-15b	.M="Missing"
.N="Not Applicable"
100="T1"
110="T1a"
120="T1b"
130="T1c"
200="T2"
210="T2a"
220="T2b"
230="T2c"
300="T3"
310="T3a"
320="T3b"
330="T3c"
999="TX"	
01. Principal	ovar_stage	Ovarian Stage (AJCC 5th Edition)		.M="Missing"
.N="Not Applicable"
100="Stage I"
110="Stage IA"
120="Stage IB"
130="Stage IC"
200="Stage II"
210="Stage IIA"
220="Stage IIB"
230="Stage IIC"
300="Stage III"
310="Stage IIIA"
320="Stage IIIB"
330="Stage IIIC"
400="Stage IV"	
01. Principal	ovar_stage_7e	Ovarian Stage (AJCC 7th Edition)	Stage for ovarian, peritoneal, or fallopian tube cancer calculated using the AJCC 7th edition staging manual.

The trial collected stage using the AJCC 5th edition staging manual.  The 7th edition stage has been calculated by using the TNM components recorded for the 5th edition stage, but applying the staging algorithm from the 7th edition staging manual.	.M="Missing"
.N="Not Applicable"
100="Stage I"
110="Stage IA"
120="Stage IB"
130="Stage IC"
200="Stage II"
210="Stage IIA"
220="Stage IIB"
230="Stage IIC"
300="Stage III"
310="Stage IIIA"
320="Stage IIIB"
330="Stage IIIC"
400="Stage IV"	
01. Principal	ovar_stage_m	Ovarian Stage M Component	Pathologic M is preferred over clinical M.	.M="Missing"
.N="Not Applicable"
0="M0"
100="M1"
999="MX"	
01. Principal	ovar_stage_n	Ovarian Stage N Component	Pathologic N is preferred over clinical N.	.M="Missing"
.N="Not Applicable"
0="N0"
100="N1"
999="NX"	
01. Principal	ovar_stage_t	Ovarian Stage T Component	Pathologic T is preferred over clinical T.	.M="Missing"
.N="Not Applicable"
100="T1"
110="T1a"
120="T1b"
130="T1c"
200="T2"
210="T2a"
220="T2b"
230="T2c"
300="T3"
310="T3a"
320="T3b"
330="T3c"
999="TX"	
01. Principal	ovar_topography	Ovarian Cancer Topography (ICD-O-2)	DEO1-C.13
DEO2-C.15
DEO3-C.11

ICD-O-2 topography.  Valid range is C000 - C809.	" "="Missing"
"C481"="Specified parts of peritoneum"
"C482"="Peritoneum, NOS"
"C569"="Ovary"
"C570"="Fallopian tube"	
05. Permutations	ovar_seer	SEER Site Recode	SEER Program cancer classification for ICD-O-2.  This is only available for confirmed cancers.	.N="Not Applicable"
21120="Peritoneum, Omentum and Mesentary"
27040="Ovary"
27070="Other Female Genital Organs"	


 
Section 19: Treatments


Class	Variable	Label	Description	Format Text	
01. Principal	ovar_curative_chemo	Had Chemotherapy For Ovarian Cancer	Did the participant have chemotherapy as part of the initial treatment for ovarian, peritoneal, or fallopian tube cancer?	.F="No Treatment Form"
.N="Not Applicable"
0="No"
1="Yes"	
01. Principal	ovar_curative_surg	Had Curative Surgery For Ovarian Cancer	Did the participant have surgery with curative intent as part of the initial treatment for ovarian, peritoneal, or fallopian tube cancer?	.F="No Treatment Form"
.N="Not Applicable"
0="No"
1="Yes"	
01. Principal	ovar_primary_trt	Known Primary Treatment For Ovarian Cancer	What is the initial primary treatment intended to cure the participant of ovarian, peritoneal, or fallopian tube cancer?	.N="Not Applicable"
1="Surgery Only"
2="Surgery and Chemotherapy"
3="Has Treatment Form, No Known Treatment with Curative Intent"
11="Pending Treatment Form"	
01. Principal	ovar_primary_trt_days	Days Until Primary Treatment For Ovarian Cancer	Days from randomization until the first known primary treatment for ovarian, peritoneal, or fallopian tube cancer.	Numeric
.N="Not Applicable"	


 
Section 20: Pathology Images


Class	Variable	Label	Description	Format Text	
02. Principal Reserved	ovar_has_deliv_heslide_img	Has a Deliverable Ovary H&E Slide Image	Has a deliverable ovary H&E Slide image? Not all were deliverable due to duplicates or no consent.	.N="Not Applicable"
1="Yes"	
02. Principal Reserved	ovar_num_heslide_imgs	Number Of Ovary H&E Slide Images	Number of deliverable ovary H&E Slide images participant has.	Numeric
.N="Not Applicable"	


 
Section 21: Screening


Class	Variable	Label	Description	Format Text	
01. Principal	ca125_days0-5	Days Until T[X] CA-125	Days from randomization until CA-125 blood test for each year.	Numeric
.F="No Form"	
01. Principal	ca125_level0-5	T[X] CA-125 Level	CA-125 level recorded from a valid blood test for each year.

Some participants received both a CA-125 version I and CA-125 version II blood test for the same year.  If the participant had valid results for both versions of the test, this variable contains the CA-125 verison I level.  Otherwise, this is the level from whichever version of the blood test the participant received.	Numeric
.F="No Form"	
01. Principal	ca125_result0-5	Result Of T[X] CA-125	CA-125 blood test result for each year.	1="Negative"
2="Abnormal, Suspicious"
4="Inadequate Screen"
8="Not Done, Expected"
9="Not Done, Not Expected"	
01. Principal	ca125ii_level0-5	T[X] CA-125 Level Version 2	CA-125 level recorded from a valid CA-125 version II blood test for each year.	Numeric
.F="No Form"	
01. Principal	tvu_days0-3	Days Until T[X] TVU	Days from randomization until  transvaginal ultrasound (TVU) screening for each screen year.	Numeric
.F="No Form"	
01. Principal	tvu_result0-3	Result Of T[X] TVU	Transvaginal ultrasound (TVU) screening result for each year.	1="Negative"
2="Abnormal, Suspicious"
3="Abnormal, Non-Suspicious"
4="Inadequate Screen"
8="Not Done, Expected"
9="Not Done, Not Expected"	
03. Components	ca125_prot	CA-125 Protocol	Screening protocol used for CA-125 blood test.	1="Expected for T0-T3 screens only, and did not receive any later screens"
2="Expected for T0-T3 and T5 screens only, or not expected for T5 screen but received it and not the T4 screen"
3="Expected for T0-T5 screens, or actually received the T4 screen"	
03. Components	ca125_src0-5	T[X] CA-125 Test Version	Version of the CA-125 test used in the main CA-125 variables.	.F="No Form"
1="CA-125I"
2="CA-125II"	
03. Components	orem_fyro	Year Of Ovary Removal	First study year where a screening form indicates the participant's ovaries were removed.	Numeric
-1="BQ indicates both ovaries removed"
.N="Not Applicable"	


 
Section 22: Diagnostic Workup


Class	Variable	Label	Description	Format Text	
01. Principal	biopolink0-5	Was There A Biopsy Linked To The T[X] Ovarian Screen	Did the positive screen in a given screen year prompt a follow-up biopsy?
Follow-up biopsies are assessed as prompted by a screen using the link or procedure chain method.	0="No"
1="Yes"	
01. Principal	ovar_mra_stat0-5	T[X] Ovarian Medical Record Abstracting Status	Completeness of screen follow-up in response to a positive screen.

Used to distinguish betweeen when the trial knows a participant did not have follow-up, and when the trial does not have enough information to know.	0="No Positive Screen"
1="Complete Information With Procedures"
2="Complete Information With No Procedures"
3="Incomplete Information, Unknown If Procedures"	


 
Section 23: Mortality Status


Class	Variable	Label	Description	Format Text	
01. Principal	dth_days	Days Until Death	Days from randomization until date of death.	Numeric
.N="Not applicable"	
01. Principal	is_dead	Dead?	Is the participant confirmed dead?	0="Not Confirmed Dead"
1="Dead"	
01. Principal	is_dead_with_cod	Dead With Known Cause?	Is the participant confirmed dead with known cause of death?	0="Not Confirmed Dead"
1="Dead"	
01. Principal	mortality_exitage	Exit Age for Mortality	Age of the participant at death, or when last known to be alive.	Numeric	
01. Principal	mortality_exitdays	Days Until Mortality Exit	Days from randomization until mortality exit date.

This is the day of death or the day last known alive. Participants are known alive through either trial contact or by queries to NDI.	Numeric	
01. Principal	mortality_exitstat	Exit Status for Mortality	Status of the participant at mortality exit. 

For participants not confirmed dead, this is the most recent contact or NDI search indicating the participant is alive.	1="Death"
2="Last NDI/Cutoff"
3="Refusal"
4="Other"	


 
Section 24: Death Certificate Cause of Death


Class	Variable	Label	Description	Format Text	
01. Principal	d_cancersite	Death Certificate Cause of Death (From Cancer)	Underlying cause of death from cancer. 

This is a grouping of ICD-9 codes from the death certificate underlying cause of death. This grouping is based on the PLCO trial cancers of interest.	.N="Not Dead"
2="Lung"
3="Colorectal"
4="Ovarian, Peritoneal, and Fallopian Tube"
11="Pancreas"
12="Melanoma of the Skin"
14="Breast"
15="Hematopoietic"
16="Endometrial"
18="Renal"
19="Thyroid"
20="Head and Neck"
99="Other Cancer"
999="Not Cancer"	
01. Principal	d_seer_death	Underlying Cause of Death	Underlying cause of death. 

This is a grouping of ICD-9 codes for underlying causes of death from the death certificate. This grouping is based on the SEER cause of death recode format, with some modifications.	.N="Not Applicable"
2="Lung"
3="Colorectal"
4="Ovarian"
5="Peritoneal"
6="Fallopian Tube"
11="Pancreas"
12="Melanoma of the Skin"
14="Breast"
15="Hematopoietic"
16="Endometrial"
18="Renal"
19="Thyroid"
20="Head and Neck"
21060="Anus, Anal Canal, and Anorectum"
37000="Miscellaneous"
50030="Septicemia"
50050="Diabetes Mellitus"
50051="Alzheimers"
50060="Diseases of Heart"
50080="Cerebrovascular Diseases"
50120="Pneumonia and Influenza"
50130="Chronic Obstructive Pulmonary Disease and Allied Cond."
50150="Chronic Liver Disease and Cirrhosis"
50160="Nephritis, Nephrotic Syndrome and Nephrosis"
50200="Symptoms, Signs and Ill-Defined Conditions"
50300="Other death"
60000="Unnatural Death"
60001="All other endocrine and metabolic diseases and immunity disorders"
60002="All other diseases of blood and blood-forming organs"
60003="Senile and presenile organic psychotic conditions"
60004="All other psychoses"
60005="Parkinson's disease"
60009="All other diseases of respiratory system"
60010="All other noninfective gastroenteritis and colitis"

[continued...]	


 


Class	Variable	Label	Description	Format Text	
01. Principal	[...continued]

d_seer_death			[...continued]

60011="All other diseases of digestive system"
60012="All other diseases of urinary system"	
05. Permutations	d_codeath_cat	Cause of Death from Death Certificate	Categorized underlying cause of death.

This is a grouping of ICD-9 codes from the death certificate underlying cause of death. This grouping is based on official trial definitions for PLCO cancers and standard ICD-9 groupings for other causes of death. The PLCO trial assesses the ICD-9 code of 185XX as prostate cancer, 162XX as lung cancer, 153XX-154XX (except 1535X) as colorectal cancer, and 183XX as ovarian cancer.	.N="Not applicable"
2="Lung"
3="Colorectal"
4="Ovarian"
5="Peritoneal"
6="Fallopian Tube"
100="Non-PLCO Neoplasms"
200="Ischemic Heart Disease"
300="Cerebrovascular Accident"
400="Other Circulatory Disease"
500="Respiratory Illness"
600="Digestive Disease"
700="Infectious Disease"
800="Endocrine, Nutritional and Metabolic Diseases, and Immunity Disorders"
900="Diseases of the Nervous System"
1000="Accident"
1100="Other"	
05. Permutations	d_dthovar	Is Ovarian Cancer The Underlying Cause Of Death?	Is ovarian cancer the underlying cause of death? This conclusion is based on the information from the death certificate.	0="No"
1="Yes"	


 
Section 25: Final Cause of Death


Class	Variable	Label	Description	Format Text	
01. Principal	f_cancersite	Cause of Death (From Cancer)	Underlying cause of death from cancer. 

This is a grouping of ICD-9 codes for underlying causes of death from the death certificate and death review. This grouping is based on the PLCO trial cancers of interest.	.N="Not Dead"
2="Lung"
3="Colorectal"
4="Ovarian, Peritoneal, and Fallopian Tube"
11="Pancreas"
12="Melanoma of the Skin"
14="Breast"
15="Hematopoietic"
16="Endometrial"
18="Renal"
19="Thyroid"
99="Other Cancer"
999="Not Cancer"	
01. Principal	f_seer_death	Final Underlying Cause of Death	Underlying cause of death. 

This is a grouping of ICD-9 codes for underlying causes of death from the death certificate and death review. This grouping is based on the SEER cause of death recode format, with some modifications.	.N="Not Applicable"
2="Lung"
3="Colorectal"
4="Ovarian"
5="Peritoneal"
6="Fallopian Tube"
11="Pancreas"
12="Melanoma of the Skin"
14="Breast"
15="Hematopoietic"
16="Endometrial"
18="Renal"
19="Thyroid"
21060="Anus, Anal Canal, and Anorectum"
37000="Miscellaneous"
50030="Septicemia"
50050="Diabetes Mellitus"
50051="Alzheimers"
50060="Diseases of Heart"
50080="Cerebrovascular Diseases"
50120="Pneumonia and Influenza"
50130="Chronic Obstructive Pulmonary Disease and Allied Cond."
50150="Chronic Liver Disease and Cirrhosis"
50160="Nephritis, Nephrotic Syndrome and Nephrosis"
50200="Symptoms, Signs and Ill-Defined Conditions"
50300="Other death"
60000="Unnatural Death"
60001="All other endocrine and metabolic diseases and immunity disorders"
60002="All other diseases of blood and blood-forming organs"
60003="Senile and presenile organic psychotic conditions"
60004="All other psychoses"
60005="Parkinson's disease"
60009="All other diseases of respiratory system"
60010="All other noninfective gastroenteritis and colitis"
60011="All other diseases of digestive

[continued...]	


 


Class	Variable	Label	Description	Format Text	
01. Principal	[...continued]

f_seer_death			[...continued]
system"
60012="All other diseases of urinary system"	
05. Permutations	f_codeath_cat	Cause of Death	Categorized underlying cause of death.

This is a grouping of ICD-9 codes for underlying causes of death from the death certificate and death review. This grouping is based on official trial definitions for PLCO cancers and standard ICD-9 groupings for other causes of death. The PLCO trial assesses the ICD-9 code of 185XX as prostate cancer, 162XX as lung cancer, 153XX-154XX (except 1535X) as colorectal cancer, and 183XX as ovarian cancer.	.N="Not applicable"
2="Lung"
3="Colorectal"
4="Ovarian"
5="Peritoneal"
6="Fallopian Tube"
100="Non-PLCO Neoplasms"
200="Ischemic Heart Disease"
300="Cerebrovascular Accident"
400="Other Circulatory Disease"
500="Respiratory Illness"
600="Digestive Disease"
700="Infectious Disease"
800="Endocrine, Nutritional and Metabolic Diseases, and Immunity Disorders"
900="Diseases of the Nervous System"
1000="Accident"
1100="Other"	
05. Permutations	f_dthovar	Is Ovarian Cancer The Underlying Cause Of Death?	Is ovarian cancer the underlying cause of death? This conclusion is based on the information from the death certificate and death review.	0="No"
1="Yes"	


 
Section 26: BQ Cohort Entry


Class	Variable	Label	Description	Format Text	
01. Principal	entryage_bq	BQ Analysis Entry Age	Age at BQ analysis entry.	Numeric	
01. Principal	entrydays_bq	BQ Analysis Entry Days	Days from the date of randomization until the participant has been randomized and completed the BQ.	Numeric	
05. Permutations	ph_any_bq	BQ Analysis Personal History of Any Cancer	Did the participant have a personal history of any cancer prior to BQ analysis entry?	0="No"
1="Yes"	
05. Permutations	ph_ovar_bq	BQ Analysis Personal History of Ovarian/Ft/Pt Cancer	Did the participant have a personal history of ovarian/ft/pf cancer prior to BQ analysis entry?	0="No"	


 
Section 27: Other Cohort Entry


Class	Variable	Label	Description	Format Text	
05. Permutations	entryage_dhq	DHQ Analysis Entry Age	Age at DHQ analysis entry.	Numeric
.F="No Form"	
05. Permutations	entryage_dqx	DQX Analysis Entry Age	Age at DQX analysis entry.	Numeric
.F="No Form"	
05. Permutations	entryage_muq	MUQ Analysis Entry Age	Age at MUQ analysis entry.	Numeric
.F="No Form"	
05. Permutations	entryage_sqx	SQX Analysis Entry Age	Age at SQX analysis entry.	Numeric
.F="No Form"	
05. Permutations	entrydays_dhq	DHQ Analysis Entry Days	Days from the date of randomization until participant has been randomized and completed the BQ and DHQ.	Numeric
.F="No Form"	
05. Permutations	entrydays_dqx	DQX Analysis Entry Days	Days from the date of randomization until participant has been randomized and completed the BQ and DQX.	Numeric
.F="No Form"	
05. Permutations	entrydays_muq	MUQ Analysis Entry Days	Days from the date of randomization until the participant has been randomized and completed the BQ and MUQ.	Numeric
.F="No Form"	
05. Permutations	entrydays_sqx	SQX Analysis Entry Days	Days from the date of randomization until the participant has been randomized and completed the BQ and SQX.	Numeric
.F="No Form"	
05. Permutations	ph_any_dhq	DHQ Analysis Personal History of Any Cancer	Did the participant have a personal history of any cancer prior to DHQ analysis entry?	.F="No Form"
0="No"
1="Yes"	
05. Permutations	ph_any_dqx	DQX Analysis Personal History of Any Cancer	Did the participant have a personal history of any cancer prior to DQX analysis entry?	.F="No Form"
0="No"
1="Yes"	
05. Permutations	ph_any_muq	MUQ Analysis Personal History of Any Cancer	Did the participant have a personal history of any cancer prior to MUQ analysis entry?	.F="No Form"
0="No"
1="Yes"	
05. Permutations	ph_any_sqx	SQX Analysis Personal History of Any Cancer	Did the participant have a personal history of any cancer prior to SQX analysis entry?	.F="No Form"
0="No"
1="Yes"	
05. Permutations	ph_ovar_dhq	DHQ Analysis Personal History of Ovarian/Ft/Pt Cancer	Did the participant have a personal history of ovarian/ft/pt cancer prior to DHQ analysis entry?	.F="No Form"
0="No"
1="Yes"	
05. Permutations	ph_ovar_dqx	DQX Analysis Personal History of Ovarian/Ft/Pt Cancer	Did the participant have a personal history of ovarian/ft/pt cancer prior to DQX analysis entry?	.F="No Form"
0="No"	
05. Permutations	ph_ovar_muq	MUQ Analysis Personal History of Ovarian/Ft/Pt Cancer	Did the participant have a personal history of ovarian/ft/pt cancer prior to MUQ analysis entry?	.F="No Form"
0="No"
1="Yes"	
05. Permutations	ph_ovar_sqx	SQX Analysis Personal History of Ovarian/Ft/Pt Cancer	Did the participant have a personal history of ovarian/ft/pt cancer prior to SQX analysis entry?	.F="No Form"
0="No"
1="Yes"	


 
Section 28: Cohort Eligibility


Class	Variable	Label	Description	Format Text	
05. Permutations	ovar_eligible_bq	BQ Ovarian/Ft/Pt Analysis: Eligible?	Flagged participants must have completed a valid questionnaire, had no history of ovarian, fallopian tube, or peritoneal cancer prior to the trial or the questionnaire, and had time at risk for their first cancer following the questionnaire.	1="Yes"	
05. Permutations	ovar_eligible_dhq	DHQ Ovarian/Ft/Pt Analysis: Eligible?	Flagged participants must have completed a valid questionnaire, had no history of cancer prior to the trial or the questionnaire, and had time at risk for their first cancer following the questionnaire.	0="No"
1="Yes"	
05. Permutations	ovar_eligible_dqx	DQX Ovarian/Ft/Pt Analysis: Eligible?	Flagged participants must have completed a valid questionnaire, had no history of cancer prior to the trial or the questionnaire, and had time at risk for their first cancer following the questionnaire.	0="No"
1="Yes"	
05. Permutations	ovar_eligible_sqx	SQX Ovarian/Ft/Pt Analysis: Eligible?	Flagged participants must have completed a valid questionnaire, had no history of ovarian, fallopian tube, or peritoneal cancer prior to the trial or the questionnaire, and had time at risk for their first cancer following the questionnaire.	0="No"
1="Yes"	
