# Supplementary material for: A lasso-based model combining miRNA and clinical variables predicts future risk of breast and ovarian cancer
Source: Sci Rep. 2026 Mar 24;16:14813. doi: 10.1038/s41598-026-45020-3 (PMC13168662; doi:10.1038/s41598-026-45020-3)
Supplement: Supplementary file 3 — Supplementary Material 3 [file 41598_2026_45020_MOESM3_ESM.pdf]

Supplementary Information

Supplementary Table 1. Full list of all 179 human miRNAs considered in this study.

Supplementary Table 2. Full list of miRNA inputted into the lasso model with corresponding lasso weights.

Supplementary Table 3. Full list of metadata variables inputted into the lasso model with corresponding lasso weights.

Supplementary Table 4. The selected $k_1 = 20$ miRNA features using lasso.

Supplementary Table 5. List of $k_2 = 5$ metadata features chosen by lasso.

Figure S1. TSNE plots of the miRNA and metadata.

Figure S2. ROC curves for *BRCA1/2* classification comparing the joint model to the metadata features alone.

Figure S3. Model performance using only the limited dataset stratified by subgroups.

Figure S4. Relation of *BRCA*ness score to breast and ovarian cancer using data from the Mass General Brigham BioBank. ROC curves are also plotted when using the *BRCA*ness score to predict ovarian/breast cancer directly in the right-hand column.

Figure S5. Schematic of classification procedure.

Figure S6. Bar charts showing the number of cases (cancer samples) and controls in each BRCA score bin for the relative risk plots in Figure 6 and Figure S4. For example, the bar chart in Figure S6(D) corresponds to the bins plotted in Figure 6(D).

Supplementary Table 1. Full list of all 179 human miRNAs considered in this study.

hsa-mir-185-5p	hsa-let-7d-5p	hsa-mir-99a-5p
hsa-mir-320e	hsa-mir-17-5p	hsa-mir-532-3p
hsa-mir-181b-5p	hsa-mir-425-5p	hsa-mir-409-3p
hsa-mir-194-5p	hsa-mir-93-5p	hsa-mir-99b-5p
hsa-mir-486-5p	hsa-mir-16-5p	hsa-mir-495-3p
hsa-mir-145-5p	hsa-mir-191-5p	hsa-mir-148b-3p
hsa-mir-422a	hsa-mir-1237-3p	hsa-mir-126-5p
hsa-mir-148a-3p	hsa-mir-26a-5p	hsa-mir-590-5p
hsa-let-7f-5p	hsa-mir-589-5p	hsa-mir-199b-3p
hsa-mir-20b-5p	hsa-mir-345-5p	hsa-mir-181a-5p
hsa-mir-199a-3p	hsa-mir-375-3p	hsa-mir-30a-5p
hsa-mir-151a-5p	hsa-let-7e-5p	hsa-mir-574-5p
hsa-mir-34c-5p	hsa-mir-28-5p	hsa-mir-378a-3p
hsa-mir-154-5p	hsa-mir-100-5p	hsa-mir-497-5p
hsa-mir-766-3p	hsa-mir-206	hsa-mir-19a-3p
hsa-mir-324-3p	hsa-mir-423-3p	hsa-mir-134-5p
hsa-mir-133b	hsa-mir-181d-5p	hsa-mir-382-5p
hsa-mir-1260a	hsa-mir-140-3p	hsa-mir-34a-5p
hsa-mir-195-5p	hsa-mir-21-5p	hsa-mir-424-5p
hsa-mir-503-5p	hsa-mir-24-3p	hsa-mir-376c-3p
hsa-mir-103a-3p	hsa-mir-210-3p	hsa-mir-222-3p
hsa-mir-92a-3p	hsa-mir-885-5p	hsa-mir-483-5p
hsa-mir-223-3p	hsa-mir-133a-3p	hsa-mir-32-5p
hsa-mir-205-5p	hsa-mir-193a-5p	hsa-let-7b-5p
hsa-mir-30e-5p	hsa-mir-320b	hsa-mir-431-3p
hsa-mir-139-3p	hsa-mir-200c-3p	hsa-mir-320d
hsa-mir-378c	hsa-mir-17-3p	hsa-mir-98-5p
hsa-mir-144-3p	hsa-mir-130a-3p	hsa-mir-15b-5p
hsa-mir-29a-3p	hsa-mir-363-3p	hsa-mir-185-3p
hsa-mir-25-3p	hsa-mir-34b-3p	hsa-mir-29b-3p
hsa-mir-584-5p	hsa-mir-33a-5p	hsa-mir-186-5p
hsa-mir-18a-5p	hsa-mir-28-3p	hsa-mir-20a-5p
hsa-mir-106b-3p	hsa-mir-142-5p	hsa-let-7g-5p
hsa-mir-487b-3p	hsa-mir-744-5p	hsa-mir-187-3p
hsa-mir-15a-5p	hsa-mir-323a-3p	hsa-mir-128-3p
hsa-mir-10b-5p	hsa-mir-30d-5p	hsa-mir-629-5p
hsa-let-7d-3p	hsa-mir-373-5p	hsa-mir-502-3p
hsa-mir-132-3p	hsa-mir-4433b-3p	hsa-mir-326
hsa-mir-328-3p	hsa-mir-708-5p	hsa-mir-130b-3p
hsa-mir-221-3p	hsa-mir-26b-5p	hsa-mir-150-5p
hsa-mir-323b-3p	hsa-mir-324-5p	hsa-mir-532-5p
hsa-mir-152-3p	hsa-mir-183-5p	hsa-mir-30c-5p
hsa-mir-103a-2-5p	hsa-mir-126-3p	hsa-mir-877-5p
hsa-mir-484	hsa-mir-770-5p	hsa-mir-501-3p
hsa-mir-106b-5p	hsa-mir-361-5p	hsa-mir-151b
hsa-mir-320c	hsa-mir-92b-3p	hsa-mir-4732-5p
hsa-mir-494-3p	hsa-let-7i-5p	hsa-mir-140-5p
hsa-mir-23a-3p	hsa-mir-454-3p	hsa-mir-27a-3p
hsa-mir-486-3p	hsa-mir-301a-3p	hsa-mir-146b-5p
hsa-mir-335-5p_0_-2	hsa-mir-421	hsa-mir-143-3p
hsa-mir-107	hsa-mir-342-3p	hsa-mir-29c-3p
hsa-mir-27b-3p	hsa-mir-196b-5p	hsa-mir-342-5p
hsa-mir-451a	hsa-mir-146a-5p	hsa-mir-660-5p
hsa-let-7c-5p	hsa-mir-181c-5p	hsa-mir-7-1-3p
hsa-mir-423-5p	hsa-mir-125b-5p	hsa-mir-18b-5p
hsa-mir-671-3p	hsa-mir-652-3p	hsa-mir-211-5p
hsa-mir-106a-5p	hsa-mir-877-3p	hsa-mir-199a-5p
hsa-mir-1260b	hsa-mir-338-3p	hsa-mir-22-5p
hsa-mir-574-3p	hsa-mir-320a-3p	hsa-mir-23b-3p
hsa-let-7a-5p	hsa-mir-122-5p	

Supplementary Table 2. Full list of miRNA inputted into the lasso model with corresponding lasso weights. The weights correspond to the normalized miRNA expression values, wherein the data are normalized as explained in the methods in the main text.

miRNA	weight	miRNA	weight	miRNA	weight
hsa-mir-185-5p	-0.417437458	hsa-let-7d-5p	0	hsa-mir-99a-5p	0.036221
hsa-mir-320e	0	hsa-mir-17-5p	-0.10674	hsa-mir-532-3p	0.163418
hsa-mir-181b-5p	-0.035306115	hsa-mir-425-5p	-0.91157	hsa-mir-409-3p	-0.04234
hsa-mir-194-5p	-0.086586402	hsa-mir-93-5p	0	hsa-mir-99b-5p	-0.07342
hsa-mir-486-5p	0.20550523	hsa-mir-16-5p	0.271739	hsa-mir-495-3p	-0.09434
hsa-mir-145-5p	0.070482152	hsa-mir-191-5p	0.601178	hsa-mir-148b-3p	0.255264
hsa-mir-422a	0.062797141	hsa-mir-1237-3p	-0.13711	hsa-mir-126-5p	0
hsa-mir-148a-3p	0.01686152	hsa-mir-26a-5p	-0.37161	hsa-mir-590-5p	0.199268
hsa-let-7f-5p	0.350975077	hsa-mir-589-5p	0.031817	hsa-mir-199b-3p	-0.00903
hsa-mir-20b-5p	0	hsa-mir-345-5p	0	hsa-mir-181a-5p	0.070877
hsa-mir-199a-3p	0.372019393	hsa-mir-375-3p	-0.05321	hsa-mir-30a-5p	0.091629
hsa-mir-151a-5p	0	hsa-let-7e-5p	-0.33449	hsa-mir-574-5p	0.04169
hsa-mir-34c-5p	0.145610024	hsa-mir-28-5p	0.047646	hsa-mir-378a-3p	0.253179
hsa-mir-154-5p	0.051820417	hsa-mir-100-5p	0.12405	hsa-mir-497-5p	0.135516
hsa-mir-766-3p	-0.042074015	hsa-mir-206	-0.09319	hsa-mir-19a-3p	0.329743
hsa-mir-324-3p	0.040111473	hsa-mir-423-3p	0.013123	hsa-mir-134-5p	0.028418
hsa-mir-133b	0.099807224	hsa-mir-181d-5p	0.003948	hsa-mir-382-5p	0.026415
hsa-mir-1260a	-0.982566416	hsa-mir-140-3p	0.049542	hsa-mir-34a-5p	-0.38243
hsa-mir-195-5p	0.141454538	hsa-mir-21-5p	0	hsa-mir-424-5p	-0.22388
hsa-mir-503-5p	0	hsa-mir-24-3p	0.187722	hsa-mir-376c-3p	0.162969
hsa-mir-103a-3p	-0.514740006	hsa-mir-210-3p	-0.06532	hsa-mir-222-3p	0.048786
hsa-mir-92a-3p	-0.081665806	hsa-mir-885-5p	-0.08095	hsa-mir-483-5p	0

hsa-mir-223-3p	-0.859989627	hsa-mir-133a-3p	-0.07779	hsa-mir-32-5p	0.00693
hsa-mir-205-5p	-0.058629116	hsa-mir-193a-5p	0.092704	hsa-let-7b-5p	-0.761
hsa-mir-30e-5p	-0.064291108	hsa-mir-320b	-0.16201	hsa-mir-431-3p	-0.01244
hsa-mir-139-3p	-0.194330385	hsa-mir-200c-3p	-0.02749	hsa-mir-320d	0.560699
hsa-mir-378c	0.073960752	hsa-mir-17-3p	-0.09404	hsa-mir-98-5p	-0.01074
hsa-mir-144-3p	0	hsa-mir-130a-3p	0	hsa-mir-15b-5p	0.434895
hsa-mir-29a-3p	-0.000220722	hsa-mir-363-3p	-0.02232	hsa-mir-185-3p	-0.18033
hsa-mir-25-3p	-0.23482439	hsa-mir-34b-3p	-0.05141	hsa-mir-29b-3p	-0.03713
hsa-mir-584-5p	0.063811871	hsa-mir-33a-5p	-0.00793	hsa-mir-186-5p	-0.01525
hsa-mir-18a-5p	-0.094723819	hsa-mir-28-3p	0	hsa-mir-20a-5p	0.000275
hsa-mir-106b-3p	-0.353365245	hsa-mir-142-5p	0.323994	hsa-let-7g-5p	0.161243
hsa-mir-487b-3p	-0.143701529	hsa-mir-744-5p	0	hsa-mir-187-3p	0.006057
hsa-mir-15a-5p	0.058457623	hsa-mir-323a-3p	-0.02145	hsa-mir-128-3p	0
hsa-mir-10b-5p	-0.026602152	hsa-mir-30d-5p	0.292882	hsa-mir-629-5p	0.222283
hsa-let-7d-3p	0.356918579	hsa-mir-373-5p	0.012366	hsa-mir-502-3p	-0.03417
hsa-mir-132-3p	-0.042109567	hsa-mir-4433b-3p	-0.02836	hsa-mir-326	0.121917
hsa-mir-328-3p	0.449285519	hsa-mir-708-5p	-0.01685	hsa-mir-130b-3p	0.548058
hsa-mir-221-3p	-0.287311763	hsa-mir-26b-5p	-0.03176	hsa-mir-150-5p	0.069221
hsa-mir-323b-3p	0.01666128	hsa-mir-324-5p	0	hsa-mir-532-5p	-0.05883
hsa-mir-152-3p	-0.084131172	hsa-mir-183-5p	0.011579	hsa-mir-30c-5p	0
hsa-mir-103a-2-5p	-0.084583347	hsa-mir-126-3p	0.18408	hsa-mir-877-5p	-0.00053
hsa-mir-484	0.78697472	hsa-mir-770-5p	0.213173	hsa-mir-501-3p	-0.08907
hsa-mir-106b-5p	0	hsa-mir-361-5p	0.050959	hsa-mir-151b	-0.09558
hsa-mir-320c	-0.086823405	hsa-mir-92b-3p	-0.02705	hsa-mir-4732-5p	0.021716
hsa-mir-494-3p	-0.108281936	hsa-let-7i-5p	0.310345	hsa-mir-140-5p	-0.11437

hsa-mir-23a-3p	0.444405907	hsa-mir-454-3p	0.018453	hsa-mir-27a-3p	0
hsa-mir-486-3p	0.063368145	hsa-mir-301a-3p	0	hsa-mir-146b-5p	-0.34044
hsa-mir-335-5p_0_-2	0	hsa-mir-421	0	hsa-mir-143-3p	-0.29661
hsa-mir-107	0	hsa-mir-342-3p	0.009062	hsa-mir-29c-3p	-0.35346
hsa-mir-27b-3p	-0.104974096	hsa-mir-196b-5p	0.025195	hsa-mir-342-5p	0.04105
hsa-mir-451a	-0.390847028	hsa-mir-146a-5p	0.170512	hsa-mir-660-5p	0
hsa-let-7c-5p	-0.012793412	hsa-mir-181c-5p	0.066446	hsa-mir-7-1-3p	-0.08585
hsa-mir-423-5p	-0.009229986	hsa-mir-125b-5p	0.190449	hsa-mir-18b-5p	-0.06256
hsa-mir-671-3p	-0.068332047	hsa-mir-652-3p	-0.42855	hsa-mir-211-5p	0.021028
hsa-mir-106a-5p	0	hsa-mir-877-3p	-0.05526	hsa-mir-199a-5p	-0.36809
hsa-mir-1260b	0.875108286	hsa-mir-338-3p	-0.28188	hsa-mir-22-5p	-0.16755
hsa-mir-574-3p	-0.131378578	hsa-mir-320a-3p	0	hsa-mir-23b-3p	0
hsa-let-7a-5p	0	hsa-mir-122-5p	0.046538		

Supplementary Table 3. Full list of metadata variables inputted into the lasso model with corresponding lasso weights. The weights correspond to the normalized metadata variables.

Variables	weight
Smoking history	0.006967
Obesity (BMI >= 30)	0
Height	-0.01083
Parity	0
Abortions	0
Ectopic pregnancies	0.01321
Hormone replacement therapy use	-0.00362
Tubal ligation	-0.00185
Benign gynecological disease	0
Endometriosis	0.005552
Benign breast disease	0.049322
Ovarian cancer in family	0.16234
Breast cancer in family	0.12378
Coronary artery disease	0
Gall bladder problems	0

Colon polyps	0
Hypertension	-0.008083
Osteoporosis	0.0153
Diabetes mellitus	0

Supplementary Table 4. The selected $k_1 = 20$ miRNA features using lasso. We also present the mean (μ) and standard deviation (σ) log2 expression for the non-*BRCA* and *BRCA* subjects, along with fold change values, and *p*-values indicating if the difference in means is significant. The fold change is defined as the mean non-*BRCA* expression divided by the mean *BRCA* expression, e.g., 0.96 = 5.95/6.2 on row 1.

miRNA	$\mu \pm \sigma$ (non-BRCA)	$\mu \pm \sigma$ (BRCA)	fold change	p value
hsa-mir-34c-5p	5.95 ± 0.97	6.2 ± 0.93	0.96	0.009
hsa-mir-133b	6.56 ± 1.09	6.74 ± 0.9	0.97	0.05
hsa-mir-223-3p	15.4 ± 1.03	14.43 ± 1.32	1.07	< 0.001
hsa-mir-18a-5p	11.04 ± 1.21	9.86 ± 1.14	1.12	< 0.001
hsa-mir-484	13.18 ± 1.06	13.13 ± 1.17	1.004	0.712
hsa-mir-425-5p	13.26 ± 0.94	12.01 ± 1.19	1.1	< 0.001
hsa-mir-1237-3p	8.21 ± 1.31	7.1 ± 0.59	1.16	< 0.001
hsa-mir-100-5p	9.37 ± 1.3	9.58 ± 1.25	0.98	0.103
hsa-mir-133a-3p	7.33 ± 1.27	6.02 ± 1.07	1.22	< 0.001
hsa-mir-130a-3p	14.1 ± 1.28	14.39 ± 1.24	0.98	0.029
hsa-mir-770-5p	7.94 ± 0.91	8.24 ± 0.9	0.96	0.002
hsa-mir-125b-5p	5.55 ± 0.77	5.82 ± 0.86	0.95	0.003
hsa-mir-338-3p	8.38 ± 1.8	6.51 ± 1.85	1.29	< 0.001
hsa-mir-34a-5p	9.28 ± 1.25	7.87 ± 1.39	1.18	< 0.001
hsa-mir-424-5p	10.42 ± 1.87	8.54 ± 2.06	1.22	< 0.001
hsa-mir-185-3p	9.17 ± 1.19	7.3 ± 1.47	1.26	< 0.001
hsa-mir-629-5p	10.4 ± 1.39	10.13 ± 1.39	1.03	0.058
hsa-mir-130b-3p	12.71 ± 1.21	12.32 ± 1.51	1.03	0.012
hsa-mir-660-5p	11.8 ± 1.53	11.53 ± 1.64	1.02	0.106

Supplementary Table 5. List of $k_2 = 5$ metadata features chosen by lasso. Statistics for these variables are included in Table 1.

Height
Benign breast disease
Ovarian cancer in family (first degree)
Breast cancer in family (first degree)
Osteoporosis (choice=yes)

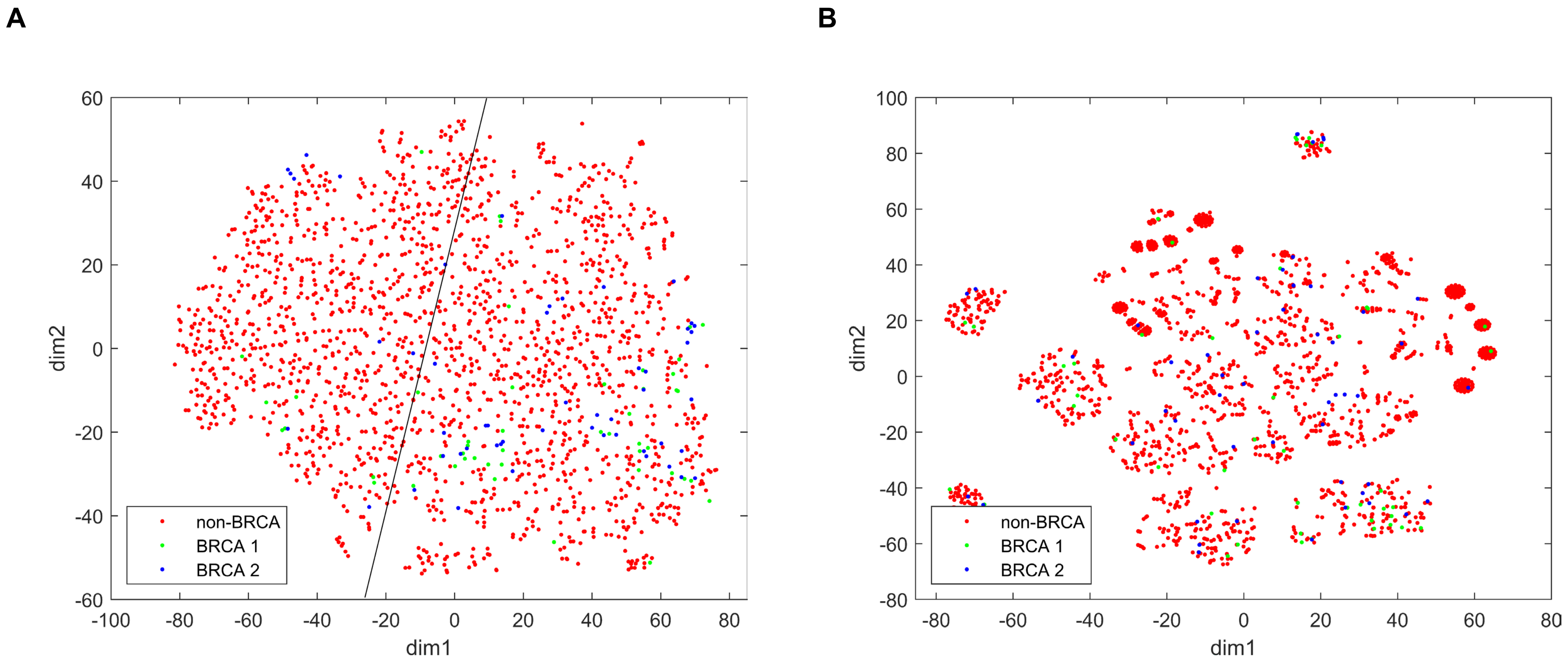

Figure S1. TSNE plots of the A) miRNA and B) metadata show the distribution of non-*BRCA*, *BRCA1*, and *BRCA2* subjects. The plot in a) shows significant linear separation between non-*BRCA* and *BRCA* (either 1 or 2) subjects in the miRNA data. There is little separation between *BRCA1* and *BRCA2* in either plot,

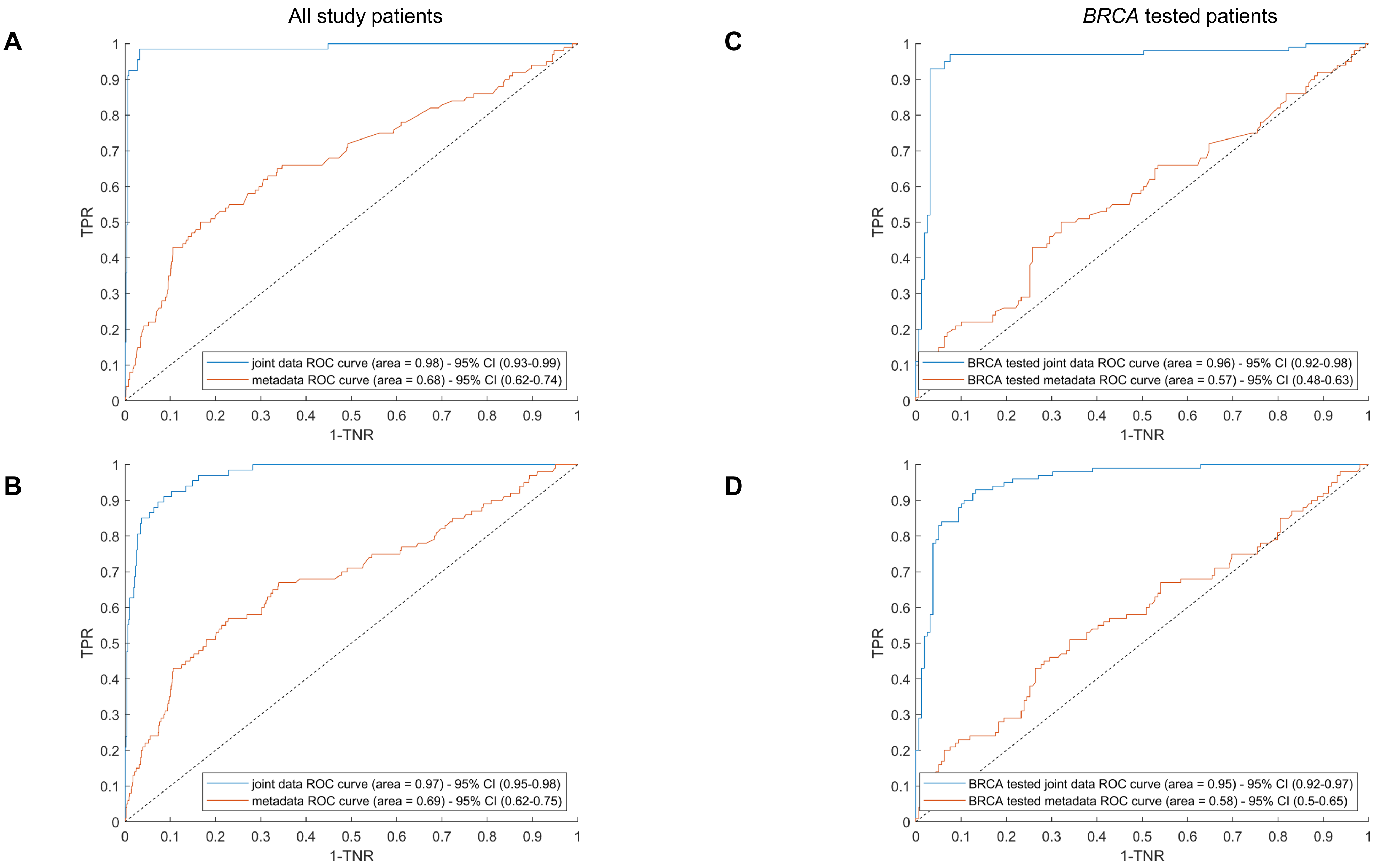

Figure S2. ROC curve for *BRCA1/2* classification comparing the joint model to the metadata features alone using A) the full dataset or B) the more limited dataset in all subjects. Examining only subjects with confirmed *BRCA* status using C) full dataset and D) limited dataset.

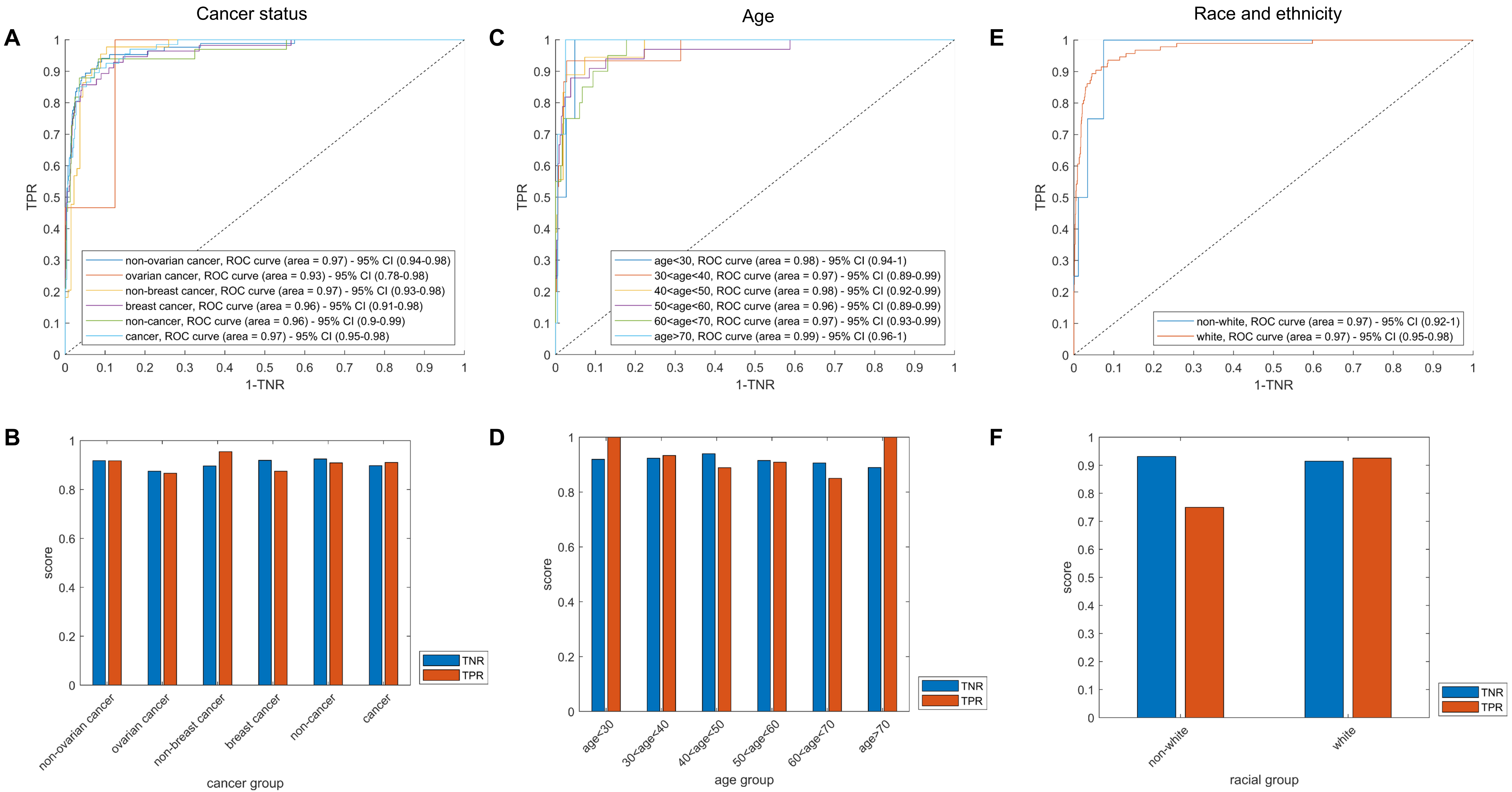

Figure S3. Model performance using only the limited dataset stratified by a) cancer history (first column); b) age (second column); and c) race/ethnicity (third column) subgroups. Top row - ROC plots. Bottom row - sensitivity (TPR) and specificity (TNR) scores among each subgroup using the t = 0.04 *BRCA* probability threshold as in Figure 1.

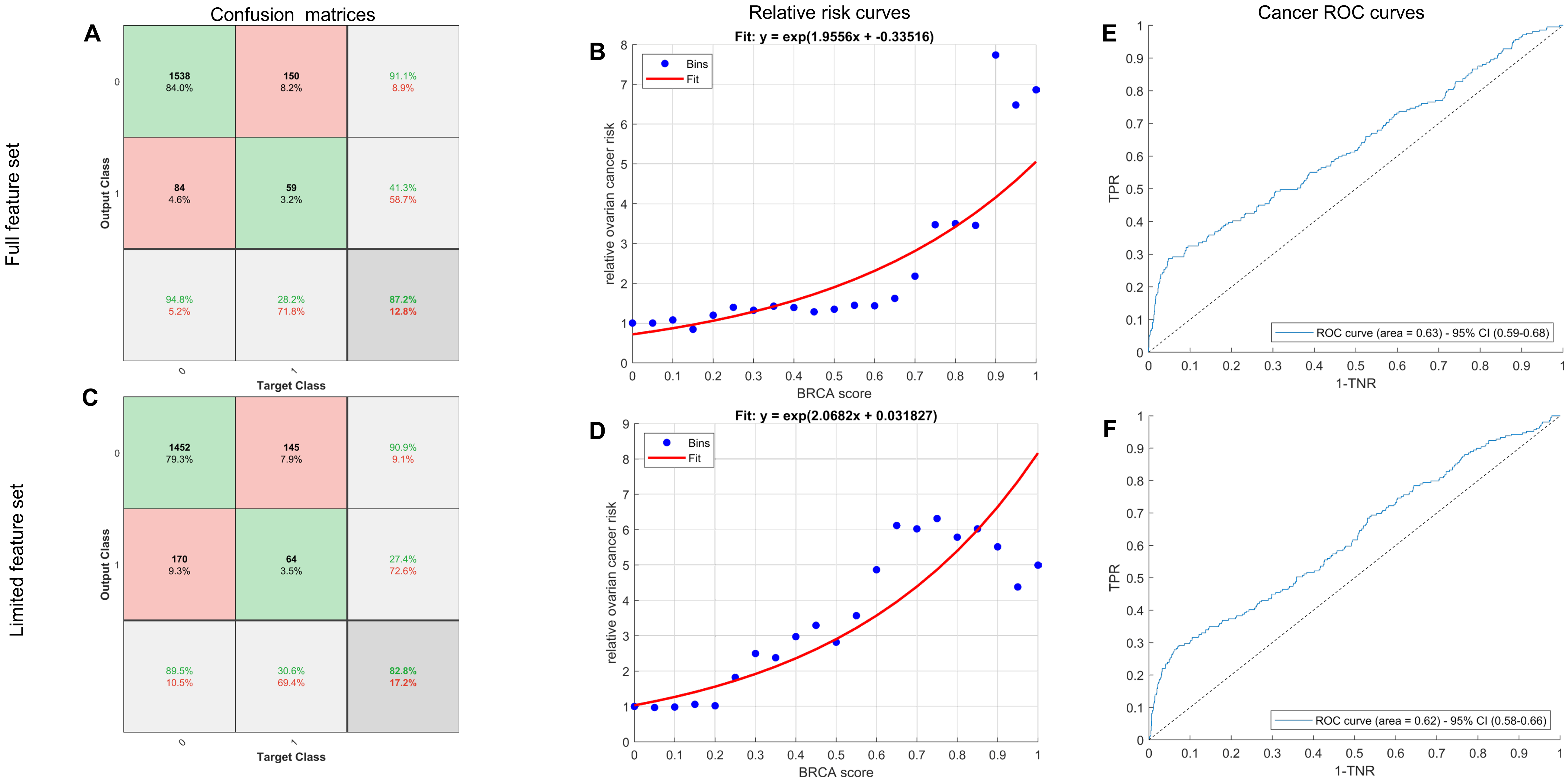

Figure S4. Relation of *BRCA*ness score to breast and ovarian cancer. A) confusion matrix when the *BRCA* model is used to predict breast and ovarian cancer directly using the Youden index on the training ROC curve. The breast and ovarian cancer subjects are considered collectively as cases and the controls are those subjects without breast or ovarian cancer. B) plot showing how relative risk of breast/ovarian cancer increases with *BRCA*ness score ($\mathrm{R} = 0.90, 95\%\ \mathrm{CI}\ 0.76 - 0.96, \mathrm{p} < 0.0001$). C) and D) show analogous results to A) and B), respectively, but using the limited data model. The log *BRCA*ness score and relative risk in D) are highly correlated with $\mathrm{R} = 0.92, 95\%\ \mathrm{CI}\ 0.80 - 0.97, \mathrm{p} < 0.0001$. The ROC curves when the *BRCA*ness score is used to predict breast/ovarian cancer directly are shown in the right-hand column.

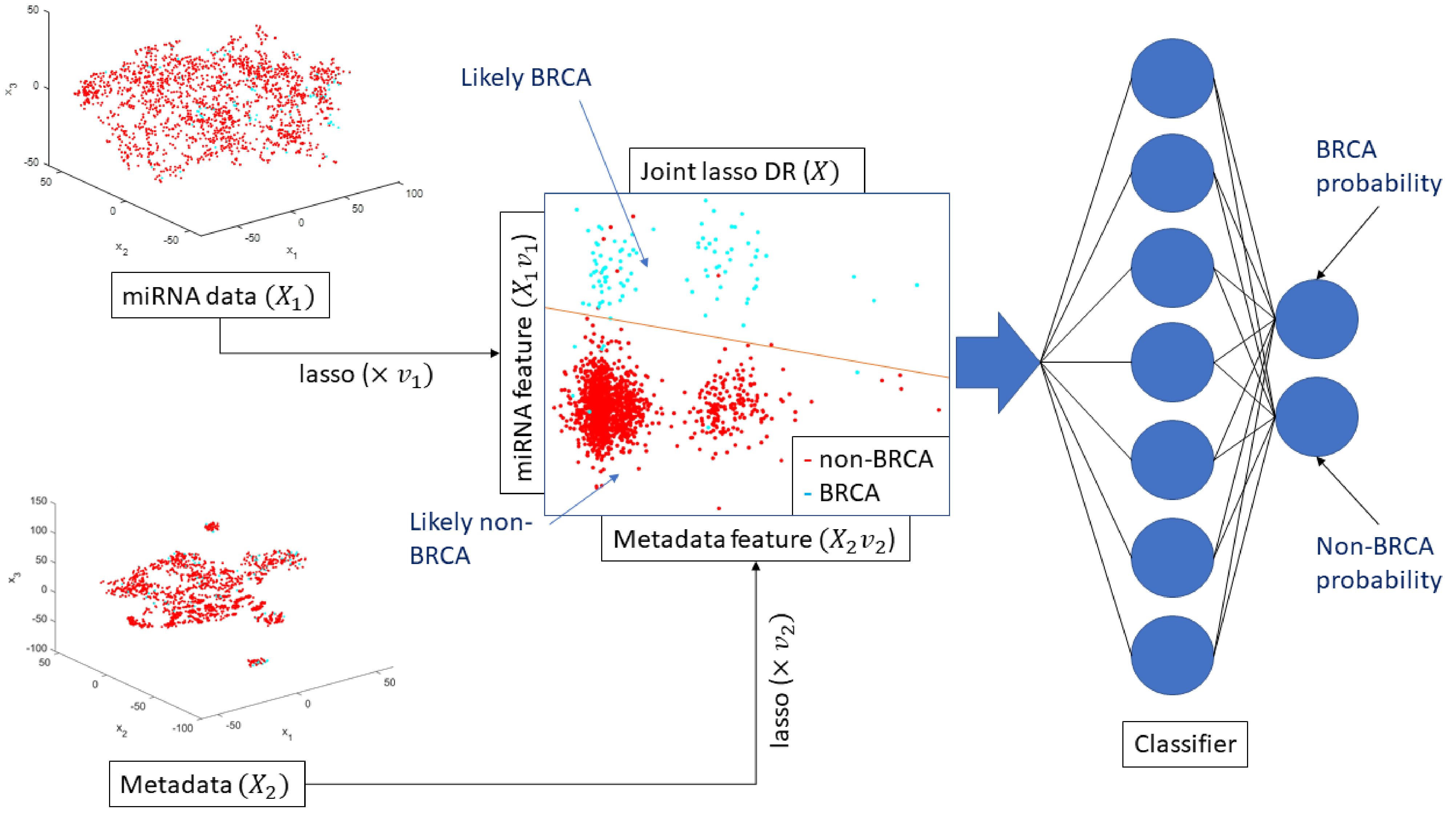

Figure S5. Schematic of classification procedure. The central figure shows the result of the joint lasso dimension reduction on the test samples after 10-fold cross validation. This is done for visualization, to show how the BRCA and non-BRCA samples separate. When the model is validated, only the training samples are used to train the classifier in the last step. x_1, x_2, and x_3 in the left-hand scatter plots denote the TSNE components.

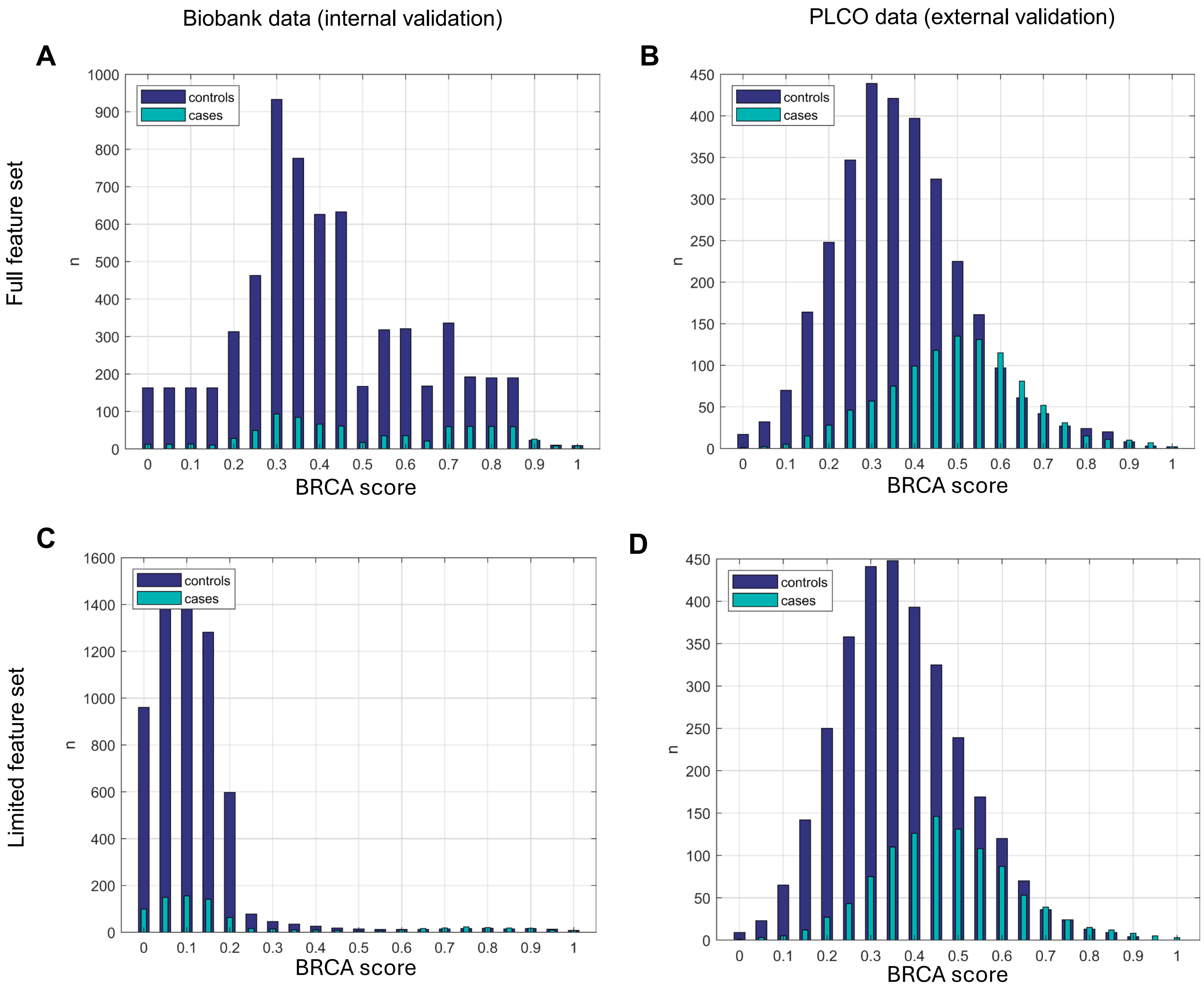

Figure S6: Bar charts showing the number of cases (cancers) and controls in each bin of the relative risk plots of Figures 6 and S4. The biobank data column corresponds to Figure S4, and the PLCO data column corresponds to Figure 6.
